# Designing Scalable Mechano‐Virucidal Nanostructured Acrylic Surfaces for Enhanced Viral Inactivation

**DOI:** 10.1002/advs.202521667

**Published:** 2026-02-13

**Authors:** Samson W. L. Mah, Denver P. Linklater, Vassil Tzanov, Chaitali Dekiwadia, Sergey Rubanov, Phuc H. Le, Laleh Tafakori, Ranya Simons, Graeme Moad, Soichiro Saita, Takashi Yanagishita, Hideki Masuda, Vladimir Baulin, Natalie A. Borg, Elena P. Ivanova

**Affiliations:** ^1^ School of Health and Biomedical Sciences RMIT University Bundoora Victoria Australia; ^2^ CSIRO Manufacturing Clayton Victoria Australia; ^3^ School of Science STEM College RMIT University Melbourne Victoria Australia; ^4^ Department of Biomedical Engineering Graeme Clarke Institute The University of Melbourne Melbourne Victoria Australia; ^5^ Bio21 Institute Ian Holmes Imaging Centre The University of Melbourne Melbourne Victoria Australia; ^6^ Departament De Química Física i Inorgànica Universitat Rovira i Virgili Tarragona Spain; ^7^ RMIT Microscopy and Microanalysis Facility STEM College RMIT University Melbourne Victoria Australia; ^8^ Mitsubishi Chemical Co. Innovation Strategy Division Chiyoda‐ku Tokyo Japan; ^9^ Department of Applied Chemistry School of Engineering Tokyo Metropolitan University Hachioji Tokyo Japan

**Keywords:** antiviral nanostructured surfaces, environmentally safe materials, mechano‐virucidal effects, nanoimprint lithography, polymers, scalable fabrication, surface mechanics

## Abstract

The transmission of viral pathogens via contaminated surfaces remains a critical public health concern, particularly in shared environments. Conventional antiviral coatings incorporating biocidal compounds face limitations due to cytotoxicity, environmental persistence, degradation, and the risk of promoting antiviral resistance. Nanostructured mechano‐bactericidal surfaces have proven effective in preventing bacterial colonization, motivating exploration of their antiviral potential. In this study, flexible nanostructured acrylic films with nanopillar arrays are fabricated using anodized aluminum oxide (AAO) molds and ultraviolet nanoimprint lithography (UV‐NIL), providing a scalable mechano‐virucidal platform, capable of physically rupturing viral particles. Systematic variation of nanopillar pitch and height reveals that interpillar spacing is the dominant determinant of antiviral efficacy. Dense arrays with a 60 nm pitch reduce human parainfluenza virus type 3 (hPIV‐3) infectivity by up to 1.2‐log (∼94%) within 1 h. Finite element method (FEM) simulations demonstrate that these arrays generate localized stresses exceeding the estimated ∼10 MPa rupture threshold of the viral envelope. In contrast, increasing the pitch to 100 nm results in diminished antiviral activity that is influenced by nanopillar height, while a 200 nm pitch abolishes antiviral activity. These findings offer a chemical‐free, mechano‐virucidal strategy for scalable antiviral surface protection across healthcare, consumer, and environmental applications.

## Graphical abstract

Can a surface be designed to physically break viruses? This study explores how nanoscale geometry—specifically the spacing of tiny pillars—can determine whether viruses remain intact or rupture. Using flexible acrylic and a scalable fabrication process, the authors develop nanopillared, transparent surfaces that show strong antiviral activity without chemicals, offering exciting potential for real‐world, touch‐safe materials.

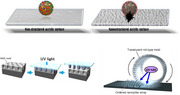

## 1 Introduction

Surface‐mediated transmission of viruses is a sufficient pathway to enable the spread of serious illness, as evidenced by the recent COVID‐19 pandemic [1, 2, 3]. Many viruses, including respiratory viruses such as SARS‐CoV‐2, respiratory syncytial virus (RSV), influenza viruses, human parainfluenza viruses (hPIVs), rhinoviruses (common cold), and enteric viruses such as noroviruses, rotaviruses, feline calicivirus, and hepatitis A virus (HAV), can be transmitted via contaminated surfaces (fomites). This is due to their ability to remain viable and infectious on surfaces for extended periods [4, 5, 6].

Efforts to minimize surface‐mediated viral transmission has driven the development of antiviral surface modification strategies, such as release‐based coatings that incorporate antiviral agents like nanoparticles, heavy metal ions, and quaternary ammonium compounds (QACs) [7, 8, 9, 10, 11, 12, 13, 14]. For example, silver, zinc, and graphene nanoparticles embedded into personal protective equipment (PPE) have been proposed, while surface coatings functionalized with natural antiviral agents such as chitosan, tannic acid, and plant‐derived resins have also been investigated [15, 16, 17, 18]. However, the reliance on active release agents raises critical concerns regarding potential cytotoxicity, environmental risks associated with leaching, and the eventual loss of antiviral efficacy as the active components are depleted [19, 20, 21, 22].

To address these challenges, contact‐based inactivation using nanotopography offers a promising alternative. Inspired by natural mechano‐bactericidal surfaces, initial studies on materials like nanospike silicon (nSi) and etched aluminum demonstrated a reduction in viral infectivity through physical disruption [23, 24, 25, 26, 27]. However, these materials are often rigid, expensive, and difficult to scale, limiting their applicability for widespread use on common objects. Polymers, by contrast, offer an ideal platform due to their low cost, light weight, and mechanical flexibility, but have remained far less explored in the antiviral context, particularly without the use of embedded antiviral additives such as nanoparticles or chemical agents [28, 29].

Among polymers, acrylics are exceptionally well‐suited for this purpose. Their inherent optical transparency, shatter‐resistance, and biocompatibility have already led to their widespread use in consumer electronics, automotive interiors, and even clinical applications like intraocular lenses, contact lenses, biomedical implants, and dental restoratives [30, 31, 32, 33, 34]. The ability to translate a mechano‐virucidal design onto such a ubiquitous and scalable material would represent a significant leap forward in infection control.

However, a key manufacturing challenge remains: creating the sub‐100 nm features necessary for interacting with viral particles in an economical, scalable manner [35]. While conventional methods like photolithography are inadequate, we have recently demonstrated that UV nanoimprint lithography (UV‐NIL) using anodized aluminum oxide (AAO) molds is a powerful technique for fabricating highly ordered nanopillar arrays on flexible polymer films [36, 37]. This approach provides the precise geometric control needed to systematically investigate the physical principles of viral inactivation.

In this study, we aimed to develop nanostructured polymer surfaces with intrinsic mechano‐antiviral activity that may help combat surface‐mediated viral transmission, while also elucidating the mechanisms by which nanoscale features disrupt viral particles. We used NIL to design regular acrylic nanoarrays of different nanopillar pitches (P) and height (H) to understand the influence of nanopattern geometry on the inactivation of the human parainfluenza virus type 3 (hPIV‐3). hPIV‐3 is a prevalent enveloped respiratory virus with significant morbidity and mortality, particularly in pediatric, elderly, and immunocompromised populations [38, 39, 40]. Viral inactivation was quantified using plaque assays, while virus–surface interactions were evaluated using advanced electron microscopy, including scanning electron microscopy (SEM), focused ion beam (FIB)–SEM , and transmission electron microscopy (TEM). The underlying mechanisms of the physical rupture of viral particles were further explored through simulation of the virus–surface interactions using COMSOL. With no current approved antivirals or vaccines available for hPIV‐3, this study provides an evidence‐based framework for designing scalable nanostructured antiviral surfaces as a practical infection control strategy.

## 2 Results

### 2.1 Fabrication and Characterization of Nanostructured Acrylic Surfaces

**FIGURE 1:**
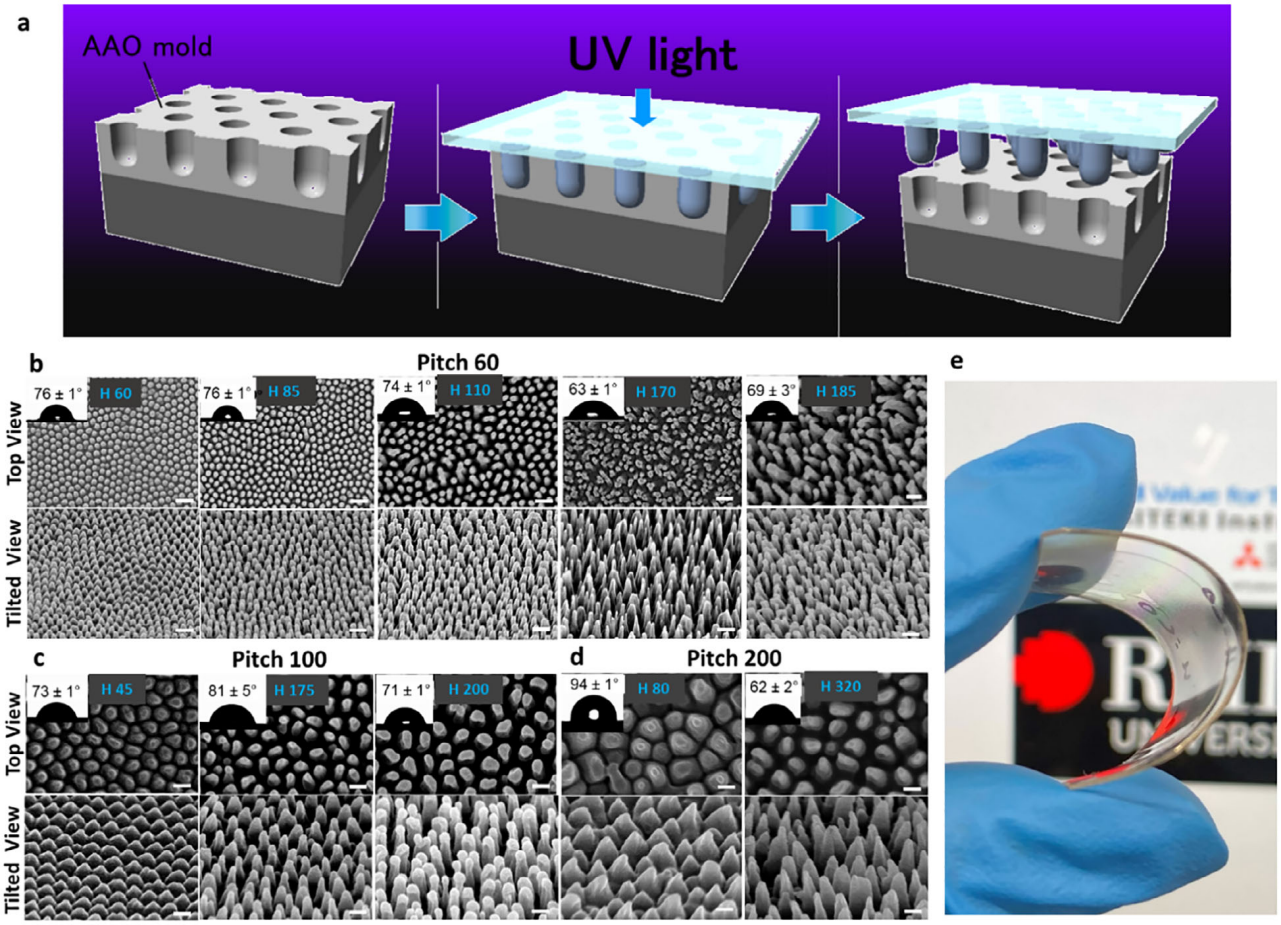
Characterization of nanofabricated acrylic surfaces with varying nanopillar pitch and height. (a) Schematic illustration of the fabrication process for nanostructured antiviral surfaces using anodized AAO molds. (b) Representative SEM images of acrylic surfaces with 60 nm pitch and height of 60, 85, 110, 170, and 185 nm. (c) Representative SEM images of 100 nm pitch and (d) 200 nm pitch with heights indicated. The upper panel of these SEM images shows the top view, while the bottom panel shows tilted views. Inset images show the corresponding water contact angle for each surface, indicating all surfaces are moderately hydrophilic. (e) Image demonstrating the bending flexibility of a nanofabricated acrylic surface. All scale bars in SEM images represent 100 nm.

The nanostructured acrylic surfaces were fabricated using a porous anodized aluminum (AAO) mold in conjunction with UV‐NIL, as previously described and illustrated in Figure 1a [41]. Ideally ordered nanohole arrangements were used as the imprinting mold, ensuring precise control of the nanopillar array structure, including the nanopillar density, diameter, height, and shape. Nanopillar arrays with a pitch of 60 nm (P 60) were fabricated with heights (H) of 60, 85, 110, 170, and 185 nm; arrays with a pitch of 100 nm (P 100) were fabricated with heights of 45, 175, and 200 nm, while those with a pitch of 200 nm (P 200) had height of 80 and 320 nm. Analysis of SEM micrographs confirmed the nanopillar array structure, including nanopillar geometry, diameter, height, and shape (Figure 1). The geometric characteristics of nanopatterns are presented in Table 1. Given the critical role of pillar spacing in determining the virucidal effectiveness of the nanostructured surfaces, a comparative statistical analysis confirmed that no significant pitch variation was observed between samples with varying heights, as shown in Figure S1.

**TABLE 1:** Geometric parameters of acrylic nano‐pillared surfaces.

Sample ID	Height, H (nm)	Pitch^a^, P (nm)	Base width^b^, BW (nm)	Tip diameter^c^, TD (nm)
		P 60		
H 60	55 ± 9	58 ± 3.5	50 ± 5	8.9 ± 1
H 85	91 ± 9	55 ± 7	45 ± 5	10 ± 3
H 110	119 ± 11	60 ± 10	42 ± 5	9.8 ± 2
H 170	179 ± 13	57 ± 8	34 ± 4	10 ± 3
H 185	191 ± 10	65 ± 13	29 ± 3	8.4 ± 2
		P 100		
H 45	45 ± 2	100 ± 6	80.2 ± 6	12 ± 2
H 175	163 ± 9	99 ± 6	53.4 ± 10	9.3 ± 2
H 200	210 ± 12	93 ± 13	60.1 ± 10	18 ± 4
		P 200		
H 80	92 ± 5	201 ± 23	112 ± 11	10 ± 3
H 320	319 ± 5	199 ± 12	77 ± 2	9.8 ± 2

^a^ Pitch is measured and defined as the center‐to‐center distance between adjacent nanopillar tips.

^b^ Measured from cross‐sectional SEM images as the full width of the pillar at its base. Base width variations are a direct geometric consequence of the mold design.

^c^ Measured from top view SEM images as the diameter of the pillar at its top. Tip diameter is set by the pore opening and remains constant.

All fabricated surfaces were moderately hydrophilic with water contact angles (WCA, Figure 1, insets) below 90°, except for the P 200 H 80 surface that exhibited a WCA of 94° [37, 42]. The nanofabricated acrylic surfaces demonstrated excellent mechanical flexibility, as shown in Figure 1e. This highlights the material's ability to maintain structural integrity even when deformed, which is critical for practical applications where surface curvature or mechanical stress may occur. The Fourier Transfer Infrared Spectroscopy (FTIR) spectra (Figure S2) revealed no detectable differences in chemical composition between the non‐structured and nanofabricated acrylic surfaces; functional groups typical of acrylic (PMMA) were observed, aligning with previous reports [43, 44].

### 2.2 Effect of Acrylic Nanopillar Geometry on Virucidal Activity

**FIGURE 2:**
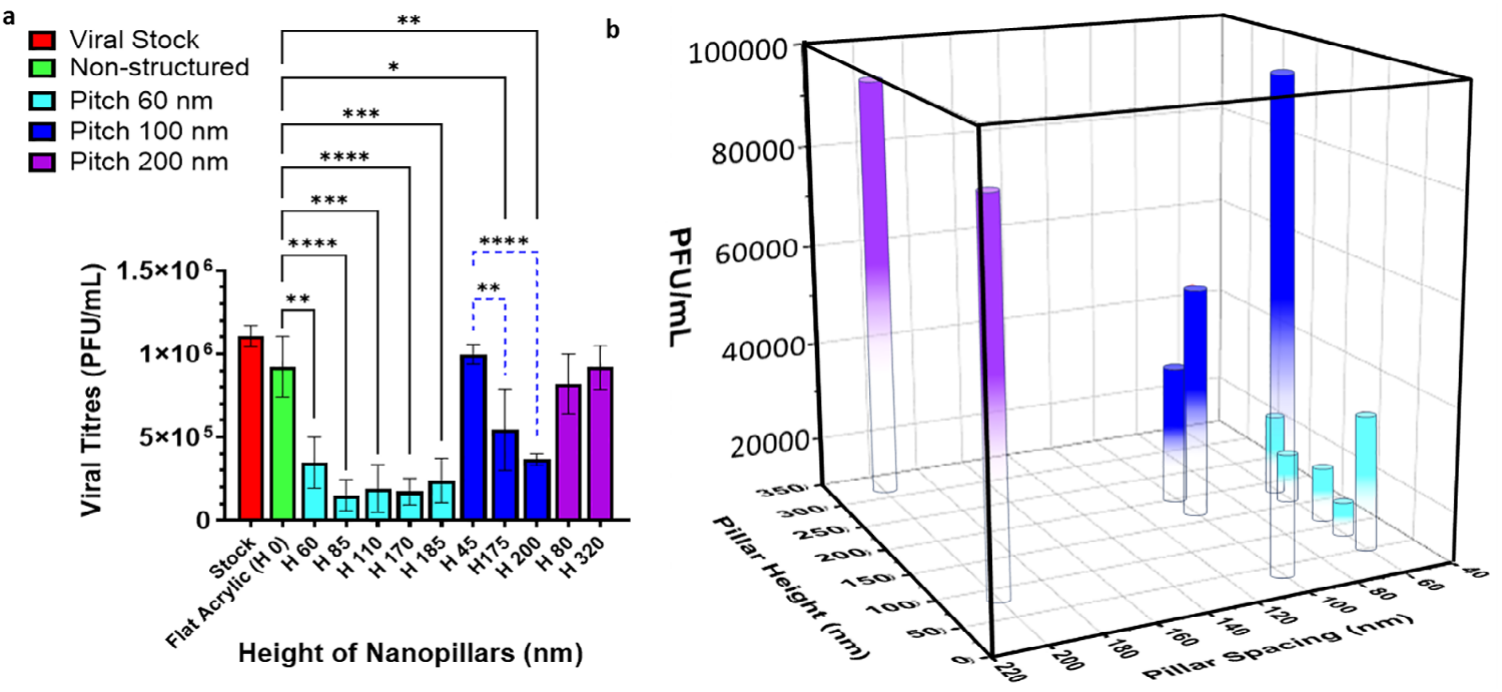
Virucidal activity of nanostructured acrylic surfaces with varying nanopillar heights and density. (a) Quantification of hPIV‐3 inactivation on acrylic surfaces with different nanopillar heights across distinct pitch groups (spacing). The recovery of infectious viral particles, measured in plaque‐forming units per milliliter (PFU/mL), is presented on the y‐axis. The ordinate values are means (± standard deviations) of at least three independent experiments. Statistical significance is indicated by symbols where shown; unlabeled pairwise comparisons are not statistically significant (*p‐value* > 0.05); ^*^, *p‐value* < 0.05; ^**^, *p‐value* < 0.01; ^***^, *p‐value* < 0.001; ^****^, *p‐value* < 0.0001. (b) 3D plot illustrating antiviral efficacy (as PFU/mL data, z‐axis) in relation to nanostructure geometry, with pillar height (nm) on the y‐axis, pitch (nm) on the x‐axis.

To evaluate the virucidal activity of the nanostructured acrylic surfaces against hPIV‐3, we conducted antiviral assays using the standard plaque assay method. The surfaces with densely packed nanopillars (P 60) significantly reduced viral infectivity, regardless of nanopillar height (ranging from 60 to 185 nm) (as shown in Figure 2a). The nanostructured acrylic surfaces reduced hPIV‐3 infectivity by an average of 0.7‐log (∼5‐fold), with the greatest reduction of 1.2‐log (∼16‐fold) observed for H 85 and H 170, relative to the non‐structured acrylic control (H 0) after just 1 h of exposure.

We further investigated the antiviral efficacy of nanopillar patterns with larger nanopillar spacings, i.e., 100 and 200 nm (P 100 and P 200, respectively), each with varying pillar height.

For intermediate spacing (P 100), pillar height played a significant role; however, our results indicated an overall decrease in virucidal efficiency at P 100 compared to nanostructured surfaces with P 60 (Figure 2). Specifically, no statistically significant reduction in viral infectivity was observed with 45 nm‐tall pillars compared to the flat control (H 0). However, increasing the pillar height to 175 nm (H 175) led to a significant increase in antiviral activity (i.e., decrease in viral titer, *p‐value* = 0.0464). The tallest pillars (H 200) at P 100 further enhanced viral reduction, achieving a 0.5‐log (∼3‐fold) decrease in infectivity (*p‐value* = 0.0022), compared to H 0.

By contrast, the least dense array with 200 nm (P 200) nanopillar spacing with either 80 or 320 nm pillars exhibited no significant antiviral activity, indicating that, at wide interpillar spacing, pillar height has little to no effect on hPIV‐3 inactivation.

We also performed reverse transcription quantitative polymerase chain reaction (RT‐qPCR) on virus samples retrieved from each P60 surface (Figures S3 and S4). The genome copy number of hPIV‐3 remained comparable across nanostructured and flat acrylic surfaces, indicating that the viral RNA was preserved. This suggests the absence of chemically mediated reactions (such as oxidation, hydrolysis, or alkylation) against the viral nucleic acid [27].

Notably, the 3D plot (Figure 2b) illustrates how nanopillar geometry affects antiviral activity. Pillar spacing has a much stronger impact than height, although at 100 nm pitch, viral titer falls linearly as the height is increased. Type II ANOVA statistical analysis revealed that pillar pitch was a highly significant determinant of viral titer (F(3,36) = 37.54, *p‐value* = 3.53 × 10^−11^), indicating that average log_10_ (PFU/mL) differed strongly among surface pitch conditions relative to flat acrylic. In addition, the Pitch × Height interaction was statistically significant (F(3,36) = 4.32, *p‐value* = 0.0106), indicating that the relationship between pillar height and viral titer is pitch‐dependent rather than uniform across all surface geometries. Thus, these results confirm that pitch has a dominant effect on the reduction in viral titer and that height modulates viral titer in a manner that depends on pitch (Detailed analysis is provided in Supplementary Statistical Analysis).

### 2.3 Interactions of hPIV‐3 Particles with Regular Nanoarrays P 60

**FIGURE 3:**
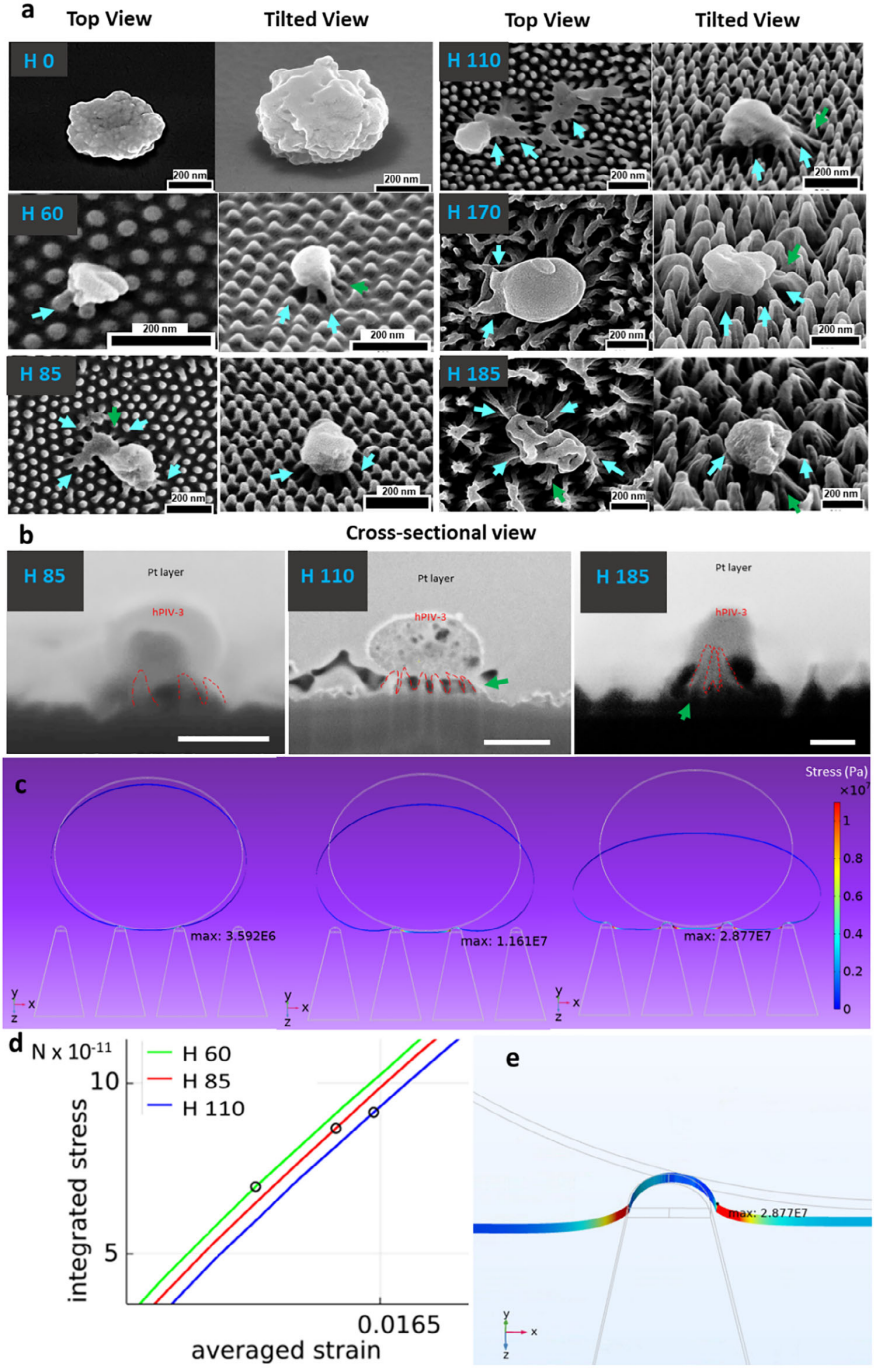
hPIV‐3 interactions with a regular array (P 60) of the nanopillars after 1 h incubation and modeling of dynamic interactions of hPIV‐3 particles and dense array (P 60) of the nanopillars. (a) SEM images showing the shape and structural characteristics of hPIV‐3 on nanopatterned surfaces. Rounded morphology is typically found on surfaces, although pleomorphism (larger average sizes and uneven shapes) of hPIV‐3 morphology can be seen, as illustrated in Figure S6. Compared to viral particles on smooth acrylic (H 0), those on nanostructured acrylic exhibit altered morphology indicative of compromised structural integrity, including envelope deformation, irregular surface features, and partial collapse. Cyan arrows highlight regions of direct virus–nanopillar interaction, capturing different stages of a dynamic mechanical engagement process. In some cases (e.g., H 85, H 110, and H 185), pronounced deformation is observed, whereas in others (e.g., H 60 and H 170), images likely represent earlier stages of interaction prior to complete morphological disruption at the time of fixation. Occasional local bending of nanopillars is also observed (dark green arrows). (b) FIB‐SEM images of the hPIV‐3—nanopillar interface showing distinct deformation of viral particles at varying pillar heights (H 85, H 110, H 185). All scale bars are 200 nm. (c) Model viral particles with a diameter of 180 nm adsorbed on a P 60 H 85 nanopillar array (see Table 1 for accurate measurements used for modelling). The maximum stress surpasses the 10 MPa damage threshold necessary for envelope rupture. (d) Stress‐strain relationship plot illustrating the critical stress points required to induce viral envelope rupturing for three nanopillar heights (H 60, H 85, and H 110) at a constant P 60, interacting with 180 nm‐diameter viral particles. Black circles denote the critical stress limit where envelope stress (at the point of maximum stress) exceeds 10 MPa. The stress and strain values are integrated over half of the viral envelope that contacts the nanopillars. (e) Zoomed‐in view of panel (c) at the point of maximum stress imposed on viral particles, providing a closer look at the stress concentration regions responsible for viral inactivation.

To investigate the mechanism of hPIV‐3 inactivation on nanostructured acrylic surfaces, interactions between hPIV‐3 and the highly virucidal P 60 nanopillar arrays were examined using SEM (Figure 3a), FIB‐SEM (Figure 3b), and TEM (Figure S5).

Examination of SEM micrographs revealed altered morphology of viral particles, including a deflated appearance, which is indicative of a loss of structural integrity (Figure 3a, cyan color arrows), consistent with the observed reduction in infectivity (Figure 2a). Notably, the nanopillars appeared to bend or flex upon contact with the viral particles. In addition to flexing, the nanopillars of 185 nm (H 185) height appeared to form clusters (Figure S10). This clustering effect could contribute toward the mechanical engagement with viral particles, as it was reported that enhanced bactericidal activity was associated with high aspect ratio nanopillars [45].

To further investigate the virus–surface interface, FIB milling was performed across multiple samples (Figure 3b). Milled cross‐sections showed direct physical interactions between the acrylic nanopillars and the viral envelope. At greater pillar heights (e.g., H 110), pillars appeared to penetrate deeply into the viral particle, suggesting a larger area of structural compromise during advanced stages of interaction. Complementary ultrathin cross‐sections obtained via ultramicrotomy and imaged with TEM (Figure S5) confirmed these observations. Viral particles were visibly clamped between nanopillars, exhibiting distorted envelopes and loss of their typical spherical morphology, further supporting a mechano‐virucidal mode of action.

To evaluate the physical interactions between hPIV‐3 particles and dense nanostructured acrylic surfaces (P 60), FEM simulations were conducted using COMSOL Multiphysics. Model particles representing hPIV‐3, with diameters of 180, 140, and 100 nm, were used. These models were used to test the interactions between the virus and the nanoarrays with pitches from 20 to 150 nm to explore the potential impact of different pitch‐to‐diameter ratios (P/d) on virucidal efficacy, as described below.

For modeling the P 60 surface, we used the measured mean of tip‐to‐tip spacing of 55 nm between the nanopillars (Table 1). When the viral particle adheres to the tips of the nanopillars, the viral envelope deforms upon contact. The von Mises stress is concentrated at the points where the viral envelope begins to lift away from the nanopillar tips (Figure 3c). Indeed, the calculated maximum stresses are above 10 MPa (1.161 × 10^7^ and 2.87 × 10^7^ N/m^2^) and occurred where the viral envelope is suspended between the nanopillars. This stress is sufficient to rupture the viral envelope. We note that an ideal model where the tip is smoothly curved was adopted and used. The reason why the maximum stress is not at the region adhered to the surface of the nanopillar is that the attraction force is absent at contact points, leading to no stress within the regions of the envelope adhered to the nanopillar tips. When the virus appears at a small distance from the nanopillar, the attraction is at its maximum strength and quadratically decreases with the increase of the distance [46]. In fact, the attraction between the virus and the nanopillar at distances above 2 nm is insignificant; see Supporting Information and Figures S7 and S8.

The critical stress points on the stress‐strain plot are presented in Figure 3d, where circles highlight the strain rate required to rupture the viral envelope for each nanopillar height. The lesser the strain that led to viral inactivation (over 10 MPa at the point of maximum stress), the more effective the nanopillar surface. Further analysis for a particular surface with gradually increased Young's modulus from 1.2 to 3.6 GPa was conducted. Our analysis has shown that, in the defined range of Young's modulus, the stiffer pillars were slightly more efficient in reaching the 10 MPa inactivation threshold for viral particles (Supplementary Theoretical Modelling and Figure S8). In our model, the stress and strain were integrated along the lower half of the viral particle, where the stress itself is integrated only over areas where it exceeded 5 MPa, and the strain is averaged. Thereby, we filter out low‐stress regions unlikely to cause envelope rupture.

Our simulations indicated that shorter nanopillars (H 60) were as effective as H 85 and H 110, in agreement with our experimental observation (see Figure 2a). However, shorter nanopillars can theoretically generate insufficient stress if we consider the possibility that there is a critical nanopillar length where the virus cannot become stretched enough because it starts touching the substrate. The case of the virus touching the substrate is partly covered in the following subsection, where we study the influence of pitch, but an in‐depth analysis of the influence of height is outside the scope of the current study. Consequently, we conclude that pillar height may not be the most critical factor for designing antiviral nanostructures with a dense arrangement of nanopillars, such as P 60.

### 2.4 Influence of Interpillar Distance (Pitch) on Virucidal Efficiency

**FIGURE 4:**
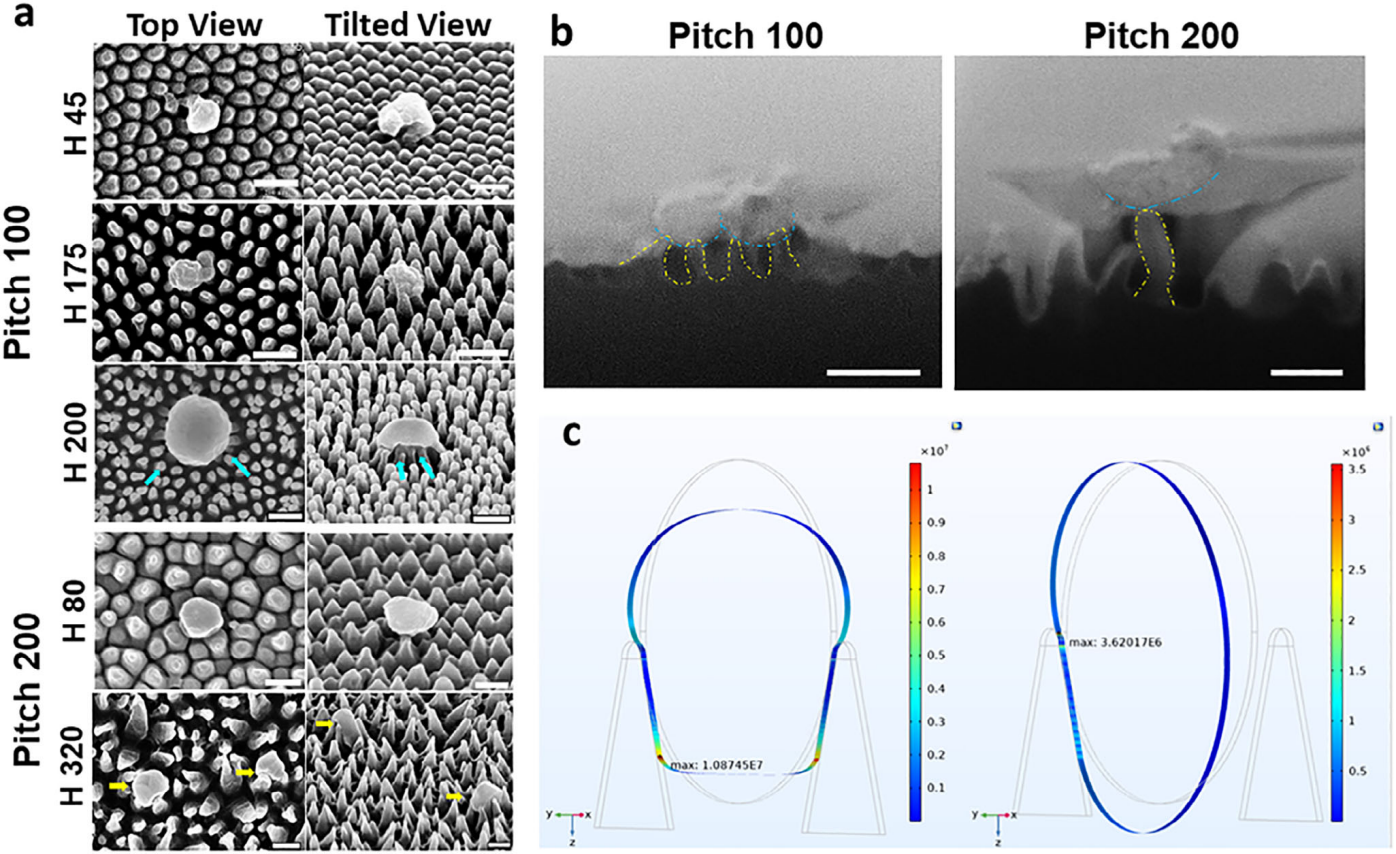
Visualization and modeling of the dynamic interactions between hPIV‐3 viral particles and P 100 and P 200 nanostructured surfaces with different nanopillar heights. Representative (a) SEM micrographs showing the interaction between hPIV‐3 and the nanopillars of different heights. Cyan arrows indicate rupture sites, while yellow arrows highlight viral particles wedged between the nanopillars. (b) FIB‐SEM micrographs of cross sections of viral particles interacting with the P 100 – H 200 (left panel) and P 200 – H 320 (right panel) surfaces at an advanced stage of their interactions. All scale bars are 200 nm. (c) FEM simulation of a modelled viral particle of 100 nm diameter interacting with the nanostructured surfaces: P/d ratio = 1.1 (left panel) and 1.2 (right panel), where P is the nanopillar pitch, and d is the diameter of the modeled viral particle. In the right panel, the threshold of 10 MPa for viral envelope rupture was not reached.

To further examine the impact of nanopillar density on virucidal activity, we investigated the antiviral efficacy of nanopillar patterns with larger nanopillar spacings, i.e., 100 and 200 nm (P 100 and P 200, respectively), each with varying pillar height. Results indicated a decrease in virucidal efficiency at a 100 nm pitch compared to nanostructured surfaces with a 60 nm pitch (Figure 2). For surfaces with shorter pillars (H 45), there was minimal virucidal activity with no significant difference observed compared to the flat control surfaces. Our experimental observations agree with the theoretical analysis, which shows that the viral particles may essentially touch the substrate, and the localized stress being exerted on the viral particles is minimal and insufficient for rupturing the viral envelope. Examination of SEM images (Figure 4a) revealed that, indeed, the viral particles are residing on nanopillars that appear flat and blunt, causing no mechanical stress. In contrast, the viral particles directly interacting with taller pillars (H 200) on surfaces with a 100 nm pitch experienced mechanical stress, leading to physical rupturing of the viral envelope as evident from both SEM and FIB‐SEM images (Figure 4a,b).

When the pitch between the nanopillars increased to 200 nm, there was no significant difference in virucidal activity compared to flat surfaces (Figure 2). The SEM images (Figure 4a, yellow arrows) revealed viral particles wedged between nanopillars, consistent with the modeling results (Figure 4c). FIB‐SEM (Figure 4b) micrographs showed minimal interaction, with only one nanopillar contacting the viral particle, resulting in insufficient localized stress to rupture the viral envelope. Thus, for the best performing surfaces of P 60 nm, the height of the nanopillars does not play a significant role, and on the surfaces with P 100 nm or P 200 nm, the antiviral activity varies slightly depending on the height of the nanopillars; however, in this case, we deem height to be irrelevant due to the low antiviral efficacy.

Furthermore, the simulations of the hPIV‐3 model interacting with nanopillar surfaces at larger pitch‐to‐diameter ratios (P/d >1) in Figure 4c demonstrated a striking difference compared to interactions when P/d values are below 1 (Figure 3c). Where P/d is equal to 1.1 (pitch = 110 nm, Figure 4c left), the simulation depicts the virus interacting with two pillars, inducing deformation that nearly reaches the 10 MPa inactivation stress threshold. Conversely, when the P/d ratio increases slightly to 1.2 (P = 120), as shown in Figure 4c (right), the virus contacts only a single pillar and can't reach the inactivation threshold, similar to what was observed in Figure 4a, P 200 (yellow arrow). Such minimal interaction between the nanopillar arrays and the viral particles means that the 10 MPa stress threshold necessary for deformation and damage of the viral envelope cannot be reached.

### 2.5 Influence of Viral Particle Diameter on Virucidal Efficiency

**FIGURE 5:**
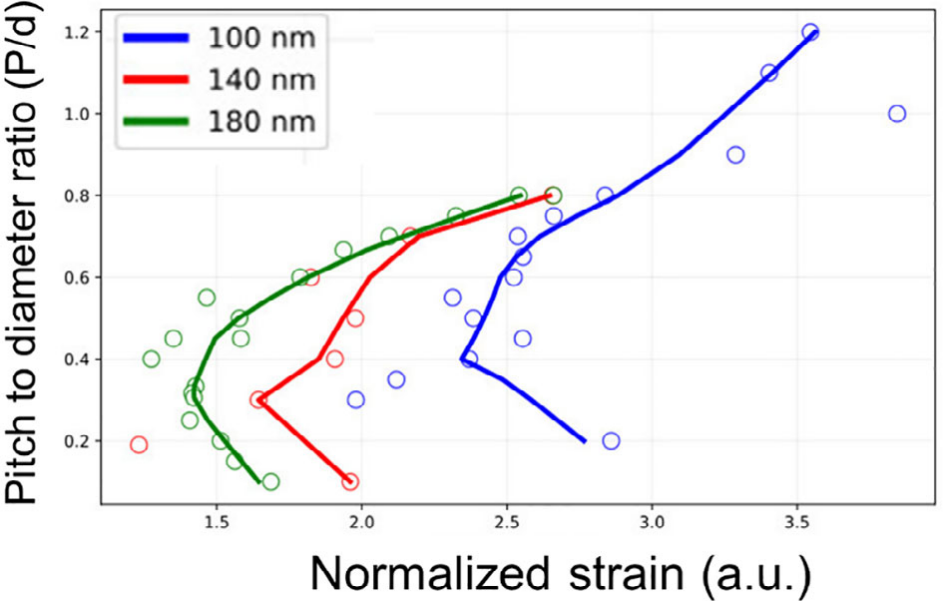
Relationship between the diameter of the viral particles and the density of the nanopillars that effectively inactivate them. The plot shows pitch to diameter (P/d) ratio versus normalized strain at the 10 MPa inactivation threshold (colored circles). Normalization accounts for simulations that overshoot 10 MPa by dividing the averaged strain by the maximum stress point value and multiplying by the value of 10^9^. Data illustrate the optimal ratio for inactivation of viral particles with diameters of 100, 140, and 180 nm.

We further investigated how the viral particle diameter (d) affects antiviral activity on nanostructured surfaces with varying nanopillar densities, using our FEM model (Figure 5). We performed FEM simulations, where a modelled viral particle represented by a sphere is gradually adsorbed onto the surface nanopillars. For each simulation, we recorded the averaged strain when the envelope stress reached the 10 MPa damage threshold [47, 48, 49]. This strain value was then divided by the corresponding maximum stress to obtain a normalized strain at inactivation, where the lower the resulting value, the more effective the virucidal surface. Our results showed that maximum virucidal efficiency occurred within a specific P/d range for a distinct diameter of the viral particle. The approximations for the viral datasets in Figure 5 were calculated using the Python LOESS package, which employs smoothing via robust locally‐weighted regression [50]. Thus, by plotting the P/d ratio against the normalized strain, we identified an optimal P/d range for hPIV‐3 particle inactivation that is between 0.2 and 0.5. This suggests that surfaces designed within this range are more likely to effectively inactivate viruses with diameters between 100 and 180 nm, consistent with the observed majority diameter distribution for hPIV‐3 (see Figure S6c).

Moreover, it appeared that for the same P/d ratio, the nanostructured surface will be more efficient toward viruses of larger diameter because the approximate curves of viral inactivation do not intersect and appear from left to right as follows: 180, 120, and 100 nm (Figure 5). The numerical results could be explained by the following. A P/d ratio of 0.3 provides fewer pillars to interact with the virus than a surface with a P/d ratio of 0.1, and the closer spacing of the 0.1 ratio allows stress distribution across more pillars, reducing overall deformation. We show in Figure 4c that larger P/d ratios can also be less effective if the virus only interacts with one pillar, preventing sufficient deformation to reach the 10 MPa stress threshold necessary for inactivation. These findings underscore the importance of optimizing P/d ratios for effective virucidal activity. This seems to be a nontrivial task, considering respiratory infectious viruses typically range from ∼30 to 200 nm in diameter. However, there is a reasonable range of P/d values from 0.2 to 0.5 that allows for virucidal nanostructured surfaces being effective toward viruses with a size distribution like hPIV‐3 (Figure S6c).

## 3 Discussion

The design of mechano‐biocidal nanostructured surfaces stands at the forefront of bio‐interface research, drawing inspiration from nature while leveraging nanoscale engineering precision [51, 52, 53]. Previous studies have established that bactericidal efficacy is largely influenced by the geometry of nanostructures [37]; particularly high aspect ratio nanostructures with increased flexibility and tight spacings exert greater stress on microorganisms [45, 54, 55, 56]. However, there is a need to understand how nanoscale geometry impacts viral inactivation. Moreover, nanostructured polymeric surfaces against viruses are understudied despite the materials being used widely in everyday products [28, 29, 57].

To investigate this, we fabricated acrylic surfaces featuring nanopillar arrays with varying spacings (P 60, P 100, and P 200) and different heights (Figure 1). We systematically tested the nanostructured acrylic surfaces for their efficacy against hPIV‐3 (150–250 nm diameter) to evaluate how nanopillar density and height influence antiviral efficacy. While surface free energy and wettability are inherently linked and can influence macroscopic adhesion, their role at the length scales relevant to viral interactions is limited [58]. Viral interaction with a surface is dominated by stochastic Brownian motion rather than classical wetting phenomena [59]. Accordingly, the moderate and comparable hydrophilicity of the tested surfaces is not expected to substantially affect virion adhesion or inactivation [60]. Instead, the antiviral response is primarily dictated by the nanoscale geometry of the nanopillar arrays.

Our findings establish a clear design principle for mechano‐virucidal polymer surfaces: nanopillar pitch is the dominant geometric factor governing antiviral efficacy. The rapid reduction of up to 1.2‐log (∼16‐fold) achieved with dense P 60 arrays, irrespective of pillar height, contrasts sharply with the height‐dependent and generally weaker activity observed for P 100 surfaces. At P 100, height becomes relevant; taller pillars can induce rupture while short pillars cannot. At P 200, height is irrelevant because spacing prevents multivalent engagement. Viruses often contact only a single pillar, producing insufficient deformation. This discovery suggests that for enveloped viruses like hPIV‐3, the mechanism is not simple piercing by individual pillars [27]. Instead, efficacy relies on the collective action of multiple, closely spaced pillars that induce critical tensile stress across the viral envelope as it attempts to settle on them. This stretching‐based mechanism, confirmed by our FEM simulations, is a critical insight for optimizing future antiviral topographies.

COMSOL FEM simulations (Figure 3d) revealed that at P 60, pillar height did not significantly impact virucidal efficiency based on stress and strain values. P 60 consistently generated von Mises stresses > 10 MPa at each investigated height. For a larger pitch, the virucidal efficacy was reduced, likely due to failure to engage multiple nanopillars simultaneously in exerting stress on the viral envelope, as seen in SEM images (Figure 4). At P 100, only taller pillars generate enough deformation to exceed the critical 10 MPa threshold for rupture, whereas shorter pillars allow the virus to contact the substrate, reducing the stress to below rupture levels. The von Mises stress generated at the virus–nanopillar interface on the surfaces with P 200 did not exceed the estimated 10 MPa rupture threshold (Supplementary Theoretical Modelling). While contact forces were present, they were insufficient to induce the mechanical stress required for envelope failure. The virucidal efficacy of the acrylic surfaces (P 60) reported in this study outperforms the antiviral efficacy of nanospike silicon (nSi) surfaces (0.8‐log reduction over 3 h) [27]. The more efficient antiviral reduction observed on acrylic surfaces compared to nSi may be attributed to greater elasticity and flexibility of the polymeric nanopillars, where the enhanced deformability allows for increased mechanical energy storage and lateral stress upon viral contact—mechanisms previously shown to amplify bactericidal effects and likely contribute to more rapid disruption of viral particles [45, 61]. Additionally, electrostatic and hydrophobic interactions may have contributed to stronger virus‐surface affinity, as supported by TEM observations (Figure S5) showing tightly associated viral particles along the nanopillar structures [62, 63].

Thus, the results of our study indicated that the interplay of the two geometric parameters of nanopillar density and height can modulate the antiviral activity. This observation implies that at intermediate spacings, the mechanical stress distribution may shift—potentially involving greater contributions from deflection‐induced lateral forces between pillars, rather than purely compressive stress directly beneath the virus [45, 54]. Besides, the measured geometrical parameters (Table 1; Figure S11) closely matched the set targets, confirming that any observed variations in antiviral efficacy are more attributable to the nanostructure design itself, rather than inconsistencies in the fabricated surface geometries. Nonetheless, the mechanical inactivation of hPIV‐3 on our nanostructured acrylic surfaces was quantitatively significant, a critical benchmark for developing surfaces capable of neutralizing airborne and surface‐transmitted viral pathogens that are usually low in their initial infectious viral titer [64, 65]. To demonstrate its dual antimicrobial potential, we further evaluated the most virucidal surface (P 60) against common bacterial strains, *Staphylococcus aureus* and *Pseudomonas aeruginosa*, which showed effective bactericidal activity (Figure S9) using methods previously described [37, 78, 79]. The antibacterial properties of the other nanostructured pitch variants have been previously reported and discussed in our earlier work [37].

Given the pleomorphic size and shape of hPIV‐3 (Figure S6), we also conducted FEM simulations using viral diameters of 100, 140, and 180 nm (Figure 5). Our findings indicate that surfaces with a (P/d) ratio between 0.2 and 0.5 consistently achieved the highest mechanical stress and virucidal efficiency. This suggests that nanostructures designed within this P/d range are optimal for compromising the viral envelope, regardless of particle size variability, while underscoring the importance of designing surfaces with nanoscale precision to ensure effective mechanical engagement with the virus.

## 4 Conclusion and Outlook

This study demonstrates that surface architecture plays a crucial role in viral disruption on soft materials and delivers great promise for the development of enhanced nanostructured antiviral surfaces. We show that pillar density is the dominant factor contributing to efficient inactivation of the hPIV‐3 virus. The acrylic nanopillar arrays with P 60 achieved reductions of up to 1.2‐log (∼94%) within just 1 h of exposure. These results contribute to a deeper understanding of how to design and improve nanostructured surfaces for effective antiviral applications. Our study establishes the critical importance of nanopillar pitch for viral inactivation and introduces a material that can be deployed across a range of industries. The FEM simulations provide a mechanistic explanation for the experimental observations and define the geometric design rules for mechano‐virucidal nanopillar surfaces. The simulations confirm that viral envelope rupture is driven by mechanical stress concentrated at points where the envelope bends between nanopillars. Dense nanopillar arrays (P 60) consistently generate von Mises stresses > 10 MPa, the estimated damage threshold for hPIV‐3's  envelope, because the viruses engage multiple pillars simultaneously, stretching the envelope between them. Stress concentrations consistently exceed the rupture threshold regardless of pillar height. Surfaces with pitch‐to‐diameter ratio P/d = 0.2–0.5 produce the strongest deformation and lowest normalized strain (i.e., highest virucidal efficiency).

The mechanical inactivation of viruses through purely physical means, coupled with the polymer's inherent flexibility, transparency, and durability, positions this nanostructured surface as a promising solution for reducing viral transmission. This approach is particularly well‐suited to non‐toxic applications, such as food packaging, electronic screens, dental restoratives, and glass replacements. Importantly, previous studies have shown that nanostructured acrylic materials can achieve high hardness, scratch resistance, and chemical resistance, and that these surfaces are inherently suited for large‐scale manufacturing [66, 67]. As shown in Figure S11, we have successfully fabricated a roll‐type translucent APA mold that enables continuous UV nanoimprint lithography for the high‐throughput generation of ordered nanopillar arrays on flexible substrates. This established and scalable process provides a practical, cost‐effective platform for the commercial deployment of functional nanostructured films [36, 68].

## 5 Experimental Section

### 5.1 Nanofabrication

The material used to fabricate acrylic film with/without nanopillars was photocurable acrylic resin PAK‐02 (Toyo Gosei., Ltd.) modified with poly(ethylene oxide) (PEO) chains as spacers. Briefly, acrylic nanopillar arrays with different pitches were produced using UV nanoimprint lithography together with a porous alumina mold fabricated by anodization of aluminum (AAO). The mold was prepared via a 2‐step anodization procedure as detailed elsewhere [69]. Prior to imprinting, the AAO mold surface was treated with a fluoroalkylsilane release agent to facilitate subsequent demolding. Briefly, liquid acrylic resin was dispensed onto the AAO mold, after which a polyethylene terephthalate (PET) film was placed on top to act as a flexible transfer substrate. The PET film ensured uniform resin spreading across the mold surface under atmospheric pressure, provided mechanical support during UV curing, and enabled clean, damage‐free delamination of the cured nanopillar film. The transfer operation occurred under atmospheric pressure for 60 s at room temperature. After pattern transfer, the acrylic underwent curing via UV irradiation (1 J / cm^2^ at 365 nm wavelength) within a nitrogen atmosphere, following established methodologies [70]. Film delamination was executed through manual peeling. To manufacture the nanopillars with different spacing, various AAO molds with corresponding pore geometries were used.

### 5.2 Surface Characterization

Acrylic surfaces were examined using the FEI Verios Scanning Electron Microscope (SEM) at 10 kV. Various magnifications ranging from ×5K to ×75K were utilized to observe the unique characteristics of the nanopillars. In instances of drifting, the acrylic samples were initially sputter‐coated before imaging, with a 10 nm layer of iridium (Ir) to enhance conductivity. Additionally, to ensure improved conductivity, the acrylic samples were mounted onto copper adhesive (Microscopy Solutions Pty Ltd, Australia) on stainless steel stubs. Image analysis and estimation of pillar characteristics and spatial distribution were conducted using ImageJ software (Version 9). At least 30 measurements were performed from 10 different SEM images, and the results are reported as mean ± standard deviation in Table 1.

### 5.3 Water Contact Angle (WCA) Measurement

Surface wettability was characterized by applying 5 µL droplets of water onto each sample. The droplet profiles were captured using a goniometer (Phoenix‐MT[T] SEO Co., Suwon, South Korea) and subsequently processed with FIJI software. Each surface's contact angle was determined as the mean of five independent measurements to ensure reproducibility.

### 5.4 FTIR

FTIR measurements were performed using a PerkinElmer Spectrum spectrometer equipped with an ATR crystal. Non‐structured and randomly selected nanostructured acrylic surfaces from each pitch group were placed directly on the ATR crystal. Spectra were recorded over the wavenumber range of 4000–400 cm^−1^ with a resolution of 4 cm^−1^. Each spectrum was averaged over 32 scans to ensure optimal signal‐to‐noise ratios. Background spectra were collected before sample measurements and automatically subtracted from the sample spectra to minimize environmental and instrumental noise. The FTIR data were analyzed [using PerkinElmer Spectrum Version 10.4.2] to identify key functional groups and evaluate changes in vibrational modes. Characteristic peaks of interest, such as those corresponding to O─H stretching, C═O stretching, or other relevant functional groups, were analysed to assess the impact of nanostructuring on the surface chemistry.

### 5.5 Cell Culture

Vero cells (ATCC CCL‐81, Manassas, USA) were recovered from liquid nitrogen and rapidly thawed in a 37°C water bath. The cell suspension was transferred into 7 mL of pre‐warmed Dulbecco's Modified Eagle Medium (DMEM; high glucose, L‐glutamine, and sodium pyruvate) supplemented with 10% heat‐inactivated fetal bovine serum (FBS) and 1% antibiotics. Following centrifugation to remove residual DMSO, the cell pellet was resuspended in fresh complete medium and seeded into cell culture flasks (Corning). Cultures were maintained at 37°C in a humidified incubator with 5% CO_2_ [71].

### 5.6 Viral Propagation and Purification

To generate working viral stock, a clinical isolate of human parainfluenza virus type‐3 (93146859, Victorian Infectious Diseases Reference Laboratory) was amplified in Vero cells. In brief, cells at ∼80% confluency were infected using 0.1 multiplicity of infection (MOI) and incubated for 1 h at 37°C with gentle agitation. The flask was then supplemented with an additional 10 mL of virus maintenance medium (DMEM + 1.6% w/v Trypsin) before further incubation at 37°C. At approximately 4–5 days, when 50% of cell death occurred, the virus‐containing medium was removed and centrifuged (3000 rcf for 15 min) to remove cell debris and stored at −80°C. For comprehensive characterization and visualization, the viral stock was first syringe‐filtered using a Millex‐HV Syringe Filter Unit, 0.45 µm, PVDF, 13 mm (Merck Millipore). Followed by purification via ultracentrifugation (80 000 rpm, 1.5 h, 4°C) through a 30% sucrose gradient cushion, and the viral pellet resuspended in PBS. The viral titer was determined by plaque assay.

### 5.7 Surface Testing

To assess the antiviral properties of the nanofabricated surfaces, 25 µL (∼10^6^ PFU/mL) of hPIV‐3 suspension was applied directly onto each test surface. Flat acrylic surfaces were used as controls. All samples, including controls, were exposed for 1 h under dark conditions at room temperature. Following incubation, the viral suspension was carefully collected. Each surface was then rinsed with 25 µL of PBS for 10 min to recover any residual viral particles. The rinse solutions were also collected. Both the retrieved viral suspensions and the PBS washes were pooled together for analysis, with half used for plaque assays and the other half for RT‐qPCR.

### 5.8 Plaque Assay

Plaque assays were performed following protocols adapted from Baer and Kehn‐Hall (2014) [72]. Vero cells were seeded in 12‐well plates at a density of approximately 250 000 cells per well and incubated overnight at 37°C in a humidified 5% CO_2_ atmosphere. The following day, the culture medium was removed, and the cells were gently washed with warm phosphate‐buffered saline (PBS). Cells were then inoculated with 200 µL of each serial dilution of the virus (in cold PBS) and incubated at 37°C for 1 h, with gentle agitation every 15 min to ensure even viral distribution. After incubation, the viral inoculum was removed, and 2.5 mL of an overlay mixture—consisting of 1.8% low‐melting‐point agarose (Lonza) in cell media supplemented with 1% penicillin‐streptomycin—was added to each well. Plates were incubated for 7 days at 37°C. To fix the monolayer, 10% formaldehyde was added directly onto the agarose in each well and left overnight. The following day, the agarose was carefully removed, and each well was stained with 500 µL of 1% crystal violet for 1 h. Wells were then rinsed with water, and viral plaques were manually counted. Viral titers were reported as plaque‐forming units per milliliter (PFU/mL).

### 5.9 Reverse Transcription Quantitative Real‐Time Polymerase Chain Reaction (RT‐qPCR)

Retrieved hPIV‐3 suspensions were subjected to RNA extraction as per the manufacturer's instructions (Isolate II RNA extraction kit (BIO‐52072, Bioline, London, UK). Followed by quantification of RNA using a nanodrop, and this was based on known base pairs and a standard formula. Serial dilutions of the extracted RNA were prepared using nuclease‐free H_2_O to achieve concentrations of 10^8^, 10^6^, 10^4^, 10^2^, and 10° copies of RNA/µL. RT‐qPCR was performed on these copies using a master mix containing TaqMan Fast Virus 1‐Step Master Mix (Thermo Fisher Cat# 4444436, Applied Biosystems, Foster City, CA, USA), custom probes (labelled fluorescently at 5’ end with 6’carboxyfluorescein [FAM] as the reporter dye and a 3’ quencher dye 6’carboxytetramethylrhodamine [TAMRA]) and primers (Thermo Fisher). Thermocycler (Quant Studio‐7, Thermo Fisher) was utilized for RT‐qPCR to determine the respective C_t_‐values (threshold crossing value) for each known 10^8^, 10^6^, 10^4^, 10^2^, and 10° copies under conditions described elsewhere [73]. A standard curve was plotted using these C_t_‐values. Subsequently, the number of genome copies of the antiviral experiment was determined by extrapolating from the generated standard curve. hPIV‐3 primers and probe sequences are provided in Figure S4.

### 5.10 Visualization of Viral Interactions

After 1 h incubation (using purified hPIV‐3 viral stock), the viruses on surfaces were initially fixed with cold 3% paraformaldehyde‐1.5% glutaraldehyde mix (PFA‐GTA) at room temperature (RT) for 1 h. Subsequently, secondary fixation was performed using 2% osmium tetroxide for 2 h at RT. Following fixation, the surfaces were rinsed thrice with distilled water before being plunged frozen into liquid nitrogen for at least 30 s. The frozen samples were then transferred into a freeze‐dryer machine for dehydration for at least 1–2 days. Once dehydrated, the surfaces were sputter‐coated with iridium and viewed under SEM under the specified conditions. For focus ion beam SEM (FIB‐SEM), samples underwent a similar primary fixation mentioned above. After primary fixation, samples underwent secondary fixation using a mixture of 2% osmium tetroxide and 1.5% potassium ferrocyanide for 1.5 h at room temperature. Following fixation, specimens were rinsed three times with distilled water and treated with 1% thiocarbohydrazide (THC) solution to enhance contrast, then washed again with water. A second exposure to 2% osmium tetroxide was applied, after which the samples were dehydrated sequentially in chilled ethanol of increasing concentration (20%, 50%, 70%, 80%, 90%, and 100%), with each step lasting 10 min. Once dehydration was complete, residual ethanol was removed, and the samples were left to dry in a biosafety cabinet for 30 min. Finally, a ∼10 nm conductive iridium coating was applied by sputtering prior to electron microscopy. During FIB‐SEM, areas of interest were identified, marked, and a 500 nm thick layer of platinum was deposited before cross‐sectional milling was performed to reveal the interface between the nanopillars and viral particles.

### 5.11 TEM

The acrylic surfaces were prepared following the FIB‐SEM protocol, but were not subjected to iridium coating. Subsequently, the surfaces were sectioned into ultrathin lateral slices, approximately 90 nm in thickness, using a Leica Ultracut Ultramicrotome (Leica Microsystems, Wetzlar, Germany) equipped with a diamond knife (Diatome, Hatfield, PA, USA). The sections were then transferred onto 200‐mesh copper grids for further analysis. Imaging was performed using a JEM 1010 transmission electron microscope (JEOL, Tokyo, Japan), and around 40 images were captured at magnifications of ×5000 and ×10 000 to facilitate detailed examination.

### 5.12 COMSOL Simulation

The Structural Mechanics module in COMSOL Multiphysics software was used to conduct finite element method (FEM) simulations, modeling the interaction between surface nanopillars and viral particles [74]. The contact interaction between the surface nanopillars and the virus is represented by a pseudo‐2D domain in the XZ‐plane that has a depth of just 2 nm and a single element along the Y‐axis. The contact pair is defined by the surfaces of the tips and the sides of the spikes, and the hemisphere of the virus that points toward the spikes. The attraction force that initiates the contact decreases quadratically with the increase of the distance, where the maximum attraction is at 0 nm (numerically shifted from 0.3 nm, see Supporting Information). Additionally, adhesion is introduced that acts on any node where the distance between the contact surfaces is less than 0.25 nm. The thickness of the adhesive layer is 0.1 nm, where the properties of the adhesive layer are the same as those of the viral envelope. The material properties of the envelope are Young's modulus E = 50 MPa, and Poisson's ratio ν = 0.3, and the ones of the acrylic nanopillars are E = 1.2 GPa and Poisson's ratio ν = 0.3 [75, 76]. When properly meshed, a series of FEM simulations were run for various shapes of the virus and spikes. The resulting output is a 2D‐model of the virus’ deformation when its envelope wraps around the nanopillars of a nanostructured surface.

As FEM cannot be perfectly tuned to simulate the forces at the nanoscale, this model is more focused on the resulting stress due to envelope deformation when the envelope follows the shape of the nanopillars. Therefore, the simulation starts with an initial value of *F*_vdw_ that defines the contact between the contact pairs (viral envelope and nanopillars), and then another simulation continues from the solution found. This repeats, and on each step, *F*_vdw_ is multiplied by a factor of 10. This way, a parametric sweep is defined where step‐by‐step, the envelope is progressively deformed along the shape of the nanopillars. Eventually, the critical stress of the viral envelope is reached at some node. Then, the averaged strain at which this occurred can be compared for the different pairs of virus envelopes and nanopillars. Therefore, we are able to compare the resulting strain of the virus’ envelope at the step when the maximum stress at a point of the envelope exceeds the critical value of 10 MPa [77]. This way, we can classify the effectiveness of a spike geometry at a given envelope by searching for the lowest strain that produces maximum point stress above 10 MPa. The relatively low Young's modulus of 50 MPa for the viral envelope and its damage threshold of 10 MPa will be further explained in Supporting Information, where we will also provide details on the contact model formulation.

### 5.13 Statistical Analysis

All quantitative data are presented as mean ± standard deviation unless otherwise stated. For geometrical analysis, at least 30 independent measurements were obtained from a minimum of 10 randomly selected SEM images per condition. Antiviral assays were performed in three independent experiments, each consisting of three individual replicates per surface. All statistical comparisons between groups were conducted using one‐way analysis of variance (ANOVA), followed by appropriate post hoc tests where applicable. The significance level of *p* < 0.05 was considered statistically significant.

Because pillar height was not measured at the same levels for each pitch and varied, the experimental design was not fully crossed. We therefore evaluated the effects of pitch, height, and their interaction using a two‐factor linear model with Type II ANOVA rather than a classical two‐way ANOVA.

Viral titers were analyzed on a logarithmic scale (log10 PFU/mL) to account for the right‐skewed and multiplicative nature of plaque‐forming unit data and to improve model assumptions. Because pillar height values were not fully crossed across pitch conditions and the flat acrylic control (Pitch = 0) was measured only at H = 0, we used a linear model that estimates pitch‐specific height dependencies rather than assuming a single global height effect. Specifically, the model was parameterized as log10 (PFU/mL) ∼ Pitch + Pitch × Height, with height mean‐centered (Hc). This approach allows direct testing of (i) differences in mean viral titer among pitch conditions and (ii) whether the effect of height on viral titer differs by pitch. Flat acrylic (H = 0) served as the reference condition. Statistical significance of model terms was assessed using Type II ANOVA, which is appropriate for unbalanced experimental designs.

## Author Contributions

S.W.L.M. designed and performed all experiments, analyzed the data, and prepared the initial manuscript draft. C.D. assisted with TEM characterization, while T.Y., S.S., and H.M. fabricated the nanostructured surfaces. FIB milling was conducted with support from D.P.L. and S.R., and AFM measurements were performed by P.H.L., V.T., and V.B. executed the COMSOL simulations and analysis. L.T. conducted a statistical analysis. E.P.I. and N.A.B. conceived the project and provided critical input throughout the study and during manuscript preparation. D.P.L., R.S., and G.M. contributed to project conceptualization, supervision, and manuscript revision. All authors discussed the results and approved the final version of the manuscript.

## Funding

This study was partly supported by the Australian Research Council by the ARC Industrial Transformational Training (ITTC) Centre in Surface Engineering for Advanced Materials (SEAM) (Grant ID: IC180100005) and ARC Discovery (Grant ID: DP240103271).

## Conflicts of Interest

The authors declare no conflicts of interest.

## Supporting information




**Supporting File**: advs74371‐sup‐0001‐SuppMat.docx.

## Data Availability

The data that support the findings of this study are available from the corresponding author upon reasonable request.
