# Supplementary material for: Designing Scalable Mechano‐Virucidal Nanostructured Acrylic Surfaces for Enhanced Viral Inactivation
Source: Adv Sci (Weinh). 2026 Feb 13;13(23):e21667. doi: 10.1002/advs.202521667 (PMC13104112; doi:10.1002/advs.202521667)
Supplement: Supplementary file 1 — Supporting File: advs74371‐sup‐0001‐SuppMat.docx. [file ADVS-13-e21667-s001.docx]

Supporting Information

Designing Scalable Mechano-Virucidal Nanostructured Acrylic Surfaces for Enhanced Viral Inactivation

Samson W. L. Mah^1,2,3^, Denver P. Linklater^3,4,5^, Vassil Tzanov^6^, Chaitali Dekiwadia^7^, Sergey Rubanov^5^, Phuc H. Le^3,5^, Laleh Tafakori^3^, Ranya Simons^2^, Graeme Moad^2^, Soichiro Saita^8^, Takashi Yanagishita^9^, Hideki Masuda^9^, Vladimir Baulin^6*^, Natalie A. Borg ^1*^, Elena P. Ivanova^3*^

^1^ School of Health and Biomedical Sciences, RMIT University, Bundoora, Victoria 3083, Australia

^2^ CSIRO Manufacturing, Clayton, Victoria 3168, Australia

^3^ School of Science, STEM College, RMIT University, Melbourne, Victoria 3000, Australia

^4^ Department of Biomedical Engineering, Graeme Clarke Institute, The University of Melbourne, Parkville, Victoria 3010, Australia

^5^ Ian Holmes Imaging Centre, Bio21 institute, The University of Melbourne, Parkville, Victoria 3010, Australia

^6^ Departament de Química Física i Inorgànica, Universitat Rovira i Virgili, C/ Marcel.lí Domingo s/n, Tarragona 43007, Spain

^7^ RMIT Microscopy and Microanalysis Facility, STEM College, RMIT University, Melbourne, Victoria 3000, Australia

^8^ Mitsubishi Chemical Co. Innovation Strategy Division, Palace Building 1-1, Marunouchi 1-chome, Chiyoda-ku, Tokyo, 100-8251, Japan

^9^ Department of Applied Chemistry, School of Engineering, Tokyo Metropolitan University, Hachioji, Tokyo 192-0397, Japan

*Analysis of Surface Pitch Regularity*


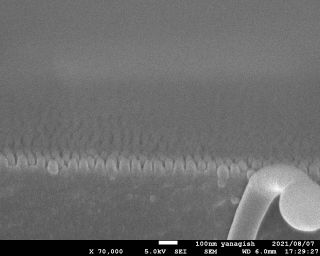

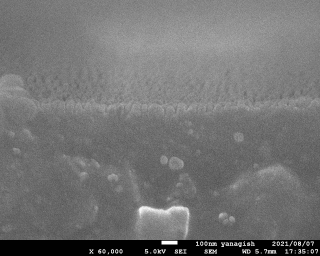

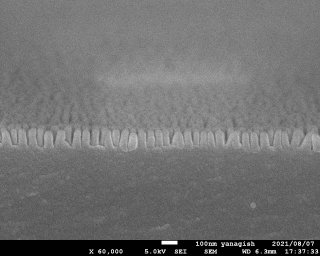

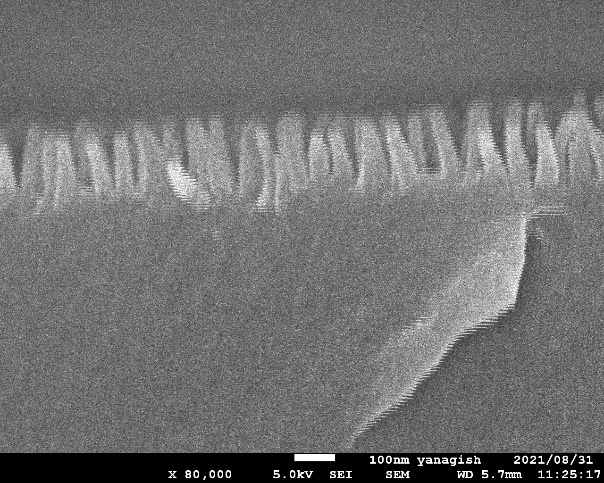

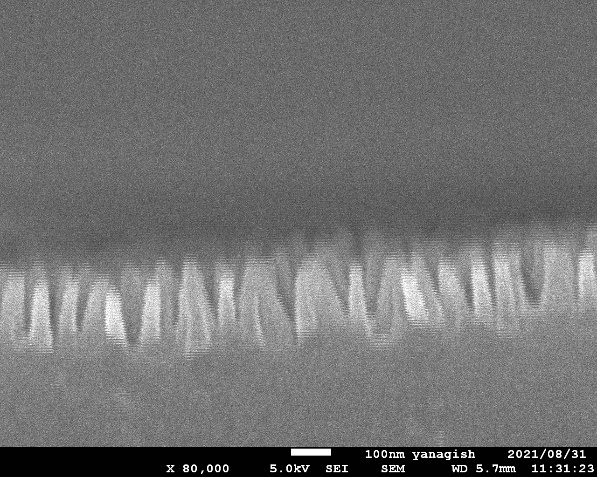


**H 60**

**H 110**

**H 85**

**H 170**

**H 185**

**a**

**b**

**Figure S1**. Surface characterization. (a) SEM images showing side view of the nanostructured acrylic surfaces P 60. (b) Graph showing statistical measurements across the similar pitch group of different heights. Each pitch group was compared across samples with different pillar heights using one-way ANOVA (n ≥ 30 per group), revealing no statistically significant differences (*p-value* > 0.05).

*Analysis of Surface’s Functional Groups*

The FTIR spectra (Figure S2 below) of the nanostructured surfaces exhibited key vibrational bands consistent with poly(methyl methacrylate) (PMMA). A strong absorption band at 1723 cm⁻¹ corresponds to the ester carbonyl (C=O) stretch, while multiple peaks in the 1140–1270 cm⁻¹ region were assigned to C–O–C stretching modes. The presence of aliphatic C–H stretching was confirmed by peaks in the 2850–2950 cm⁻¹ range, associated with the methyl and methylene groups in the PMMA backbone. Additional bending vibrations of –CH₃ groups appeared between 1380–1480 cm⁻¹. Collectively, these features validate the chemical identity of PMMA and indicate that no significant structural degradation or chemical modification occurred during the fabrication process.


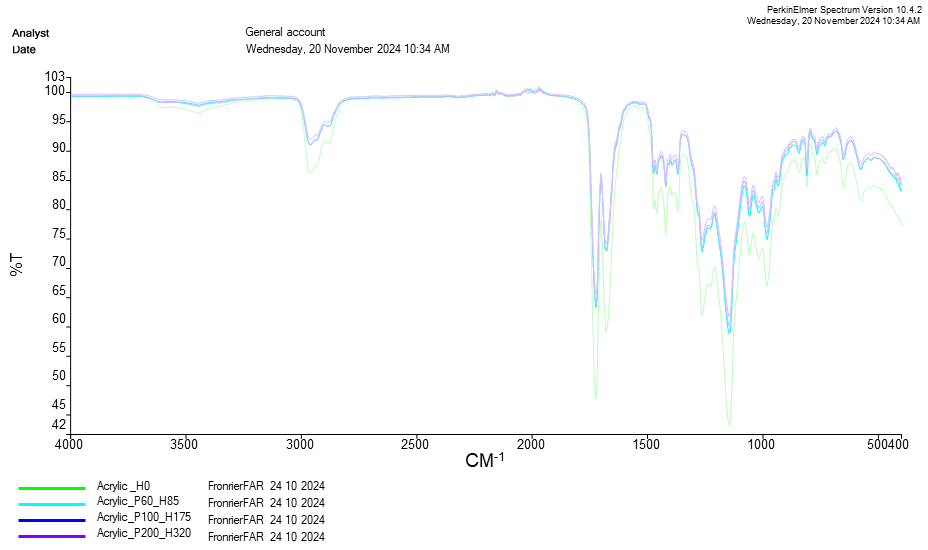


**Figure S2** FTIR spectrum of non-structured and nanostructured acrylic surfaces. The FTIR spectra showed characteristic bands for polyacrylic copolymers, with esters in the backbone chain contributing to C–C–O and C–C=O bonds (1100–1400 cm⁻¹), carbonyl (C=O) bands (1720–1725 cm⁻¹), and probably C–H vibrations (2920–2850 cm⁻¹).

*RT-qPCR*

**Figure S3.** RT-qPCR quantification of hPIV-3 genome copy number recovered from all test surfaces. No significant differences were detected, indicating consistent viral genome recovery across surfaces.). Data are presented as mean ± standard deviation from independent experiments. Statistical significance is indicated as ***** *(p-value < 0.0001), ** (p-value < 0.01), * (p-value < 0.05),* and *ns (p-value > 0.05).*

**Figure S4.** Primer and probe sequences designed for quantitative detection of human parainfluenza virus type 3 (hPIV-3) using reverse transcription quantitative polymerase chain reaction (RT-qPCR). The primers target a conserved region of the hemagglutinin-neuraminidase (HN) gene, yielding a specific amplicon for sensitive viral RNA detection. The TaqMan custom probes (labelled fluorescently at 5’ end with 6’carboxyfluorescein [FAM] as the reporter dye and a 3’ quencher dye 6’carboxytetramethylrhodamine [TAMRA]) was optimized to minimize secondary structure and primer–dimer formation. Primer and probe sequences were designed using the Integrated DNA Technologies (IDT) online design tool, ensuring high specificity for hPIV-3.

*TEM imaging of hPIV-3 and nanostructured acrylic interactions.*


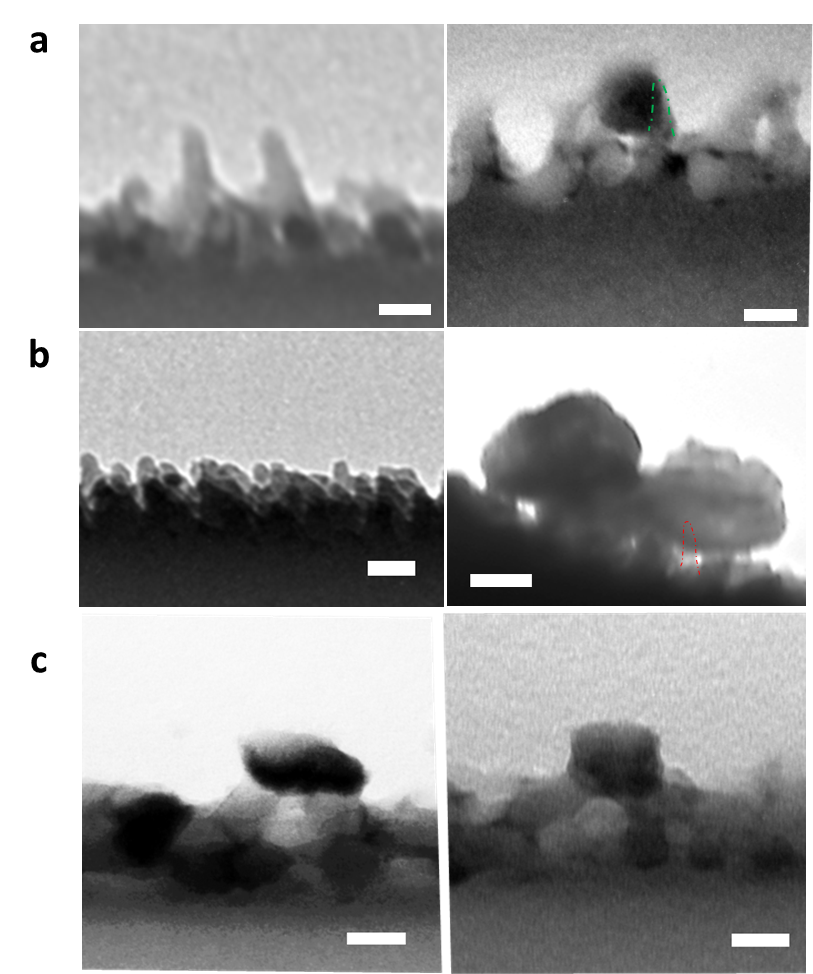


**Figure S5.** TEM images illustrate dynamic interactions between hPIV-3 and nanostructured acrylic surfaces (P60). (a) Left: intact nanopillar arrays in the absence of viral particles. Right: an early-stage interaction, where nanopillars begin to wrap around a virion; green dashed lines highlight regions of mechanical engagement that precede envelope stretching. (b) Left: zoomed-out view of the nanopillar array without virus. Right: intermediate-stage interaction showing partial invagination of nanopillars into the viral envelope; the red dashed line traces the extent of invagination relative to the undeformed nanopillars in the left panel.(c) Later-stage interactions exhibiting pronounced viral deformation, where nanopillars appear fully invaginated into the viral envelope when compared with the intact nanopillar references in left panel (a), indicating sustained mechanical stress that likely compromises viral structural integrity. All scale bars are 50 nm.

*Pleomorphism of hPIV-3*


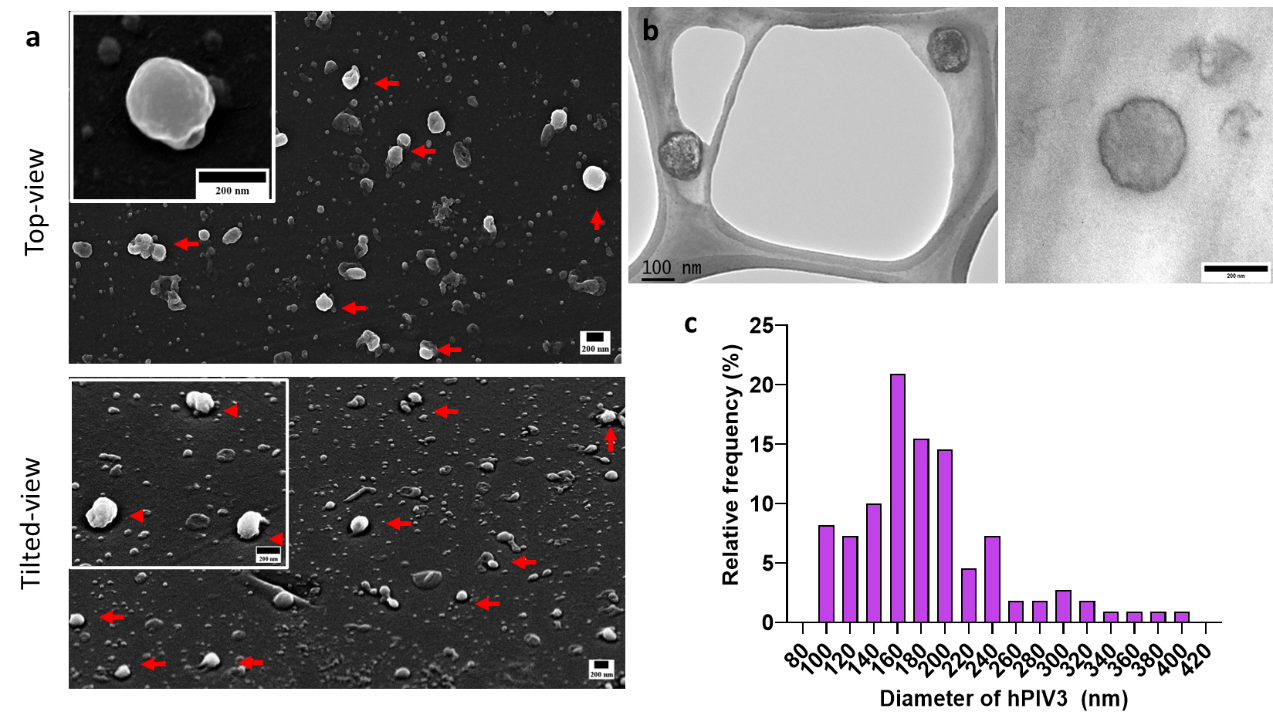


**Figure S6**. Low (a) and high (b) magnification SEM images of purified hPIV-3 fixed on non-structured acrylic surfaces. hPIV-3 were negative stained/fixed using osmium tetroxide and hence appears brighter in contrast as indicated by red arrows. hPIV3 was mainly globular in shape and appeared to be pleomorphic in size as there were much larger (>250 nm) viral particles found throughout the surfaces. All scale bars are 200 nm. (c) Nominal size distributions of hPIV-3 particles observed on non-structured surfaces.

*Supplementary Statistical Analysis*

Model coefficients clarify the magnitude and direction of pitch effects. The intercept (6.00) corresponds to the predicted mean viral titer on flat acrylic at the average height of the dataset, equivalent to approximately 1.0 × 10⁶ PFU/mL. Relative to this reference condition, Pitch 60 showed a large and highly significant reduction in viral titer (β = −0.716 log10 units, 95% CI: −0.88 to −0.55, *p-value* = 1.69 × 10⁻¹⁰), corresponding to an approximately 82% decrease in PFU/mL at the mean height. Pitch 100 also exhibited a smaller but statistically significant reduction relative to flat acrylic (β = −0.168 log10 units, 95% CI: −0.34 to −0.00004, *p-value* = 0.0499). In contrast, Pitch 200 did not differ significantly from the flat control (β = −0.088 log10 units, 95% CI: −0.28 to 0.11, *p-value* = 0.37). These results indicate that the strongest pitch-dependent suppression of viral titer occurs at the 60-nm pitch, with progressively weaker effects at larger pitches.

The significant Pitch × Height interaction reflects differences in how viral titer responds to changes in height within each pitch condition. For Pitch 60, the estimated height slope was negative but not statistically significant (−7.27 × 10⁻⁴ log10 PFU per nm, *p-value* = 0.46), indicating no strong evidence for a height-dependent effect within this pitch. In contrast, Pitch 100 exhibited a significant negative height dependence (−2.57 × 10⁻³ log10 PFU per nm, 95% CI: −4.07 × 10⁻³ to −1.08 × 10⁻³, *p-value* = 0.0013), demonstrating that viral titer decreases systematically with increasing height under this pitch condition. For example, a 100-nm increase in height at Pitch 100 corresponds to an expected decrease of approximately 0.26 log10 units, or roughly a 48% reduction in PFU/mL. Pitch 200 showed no significant height dependence (*p-value* = 0.69), indicating that within the tested range, height does not measurably affect viral titer for this pitch.

The model explained a substantial fraction of the variability in viral titers (R² = 0.78; adjusted R² = 0.74), and the overall regression was highly significant (F(6,36) = 20.93, *p-value* = 2.16 × 10⁻¹⁰).

*Supplementary Theoretical Modelling*

Our COMSOL simulations consider the attractive term of the Lennard-Jones (LJ) potential together with adhesion at sub-nanometer distance to model the interaction between the 2D - nanosphere (virus) and 2D - nanopillars interaction. The LJ potential exhibits a quadratic decline. The adhesion is activated below 0.25 nm.


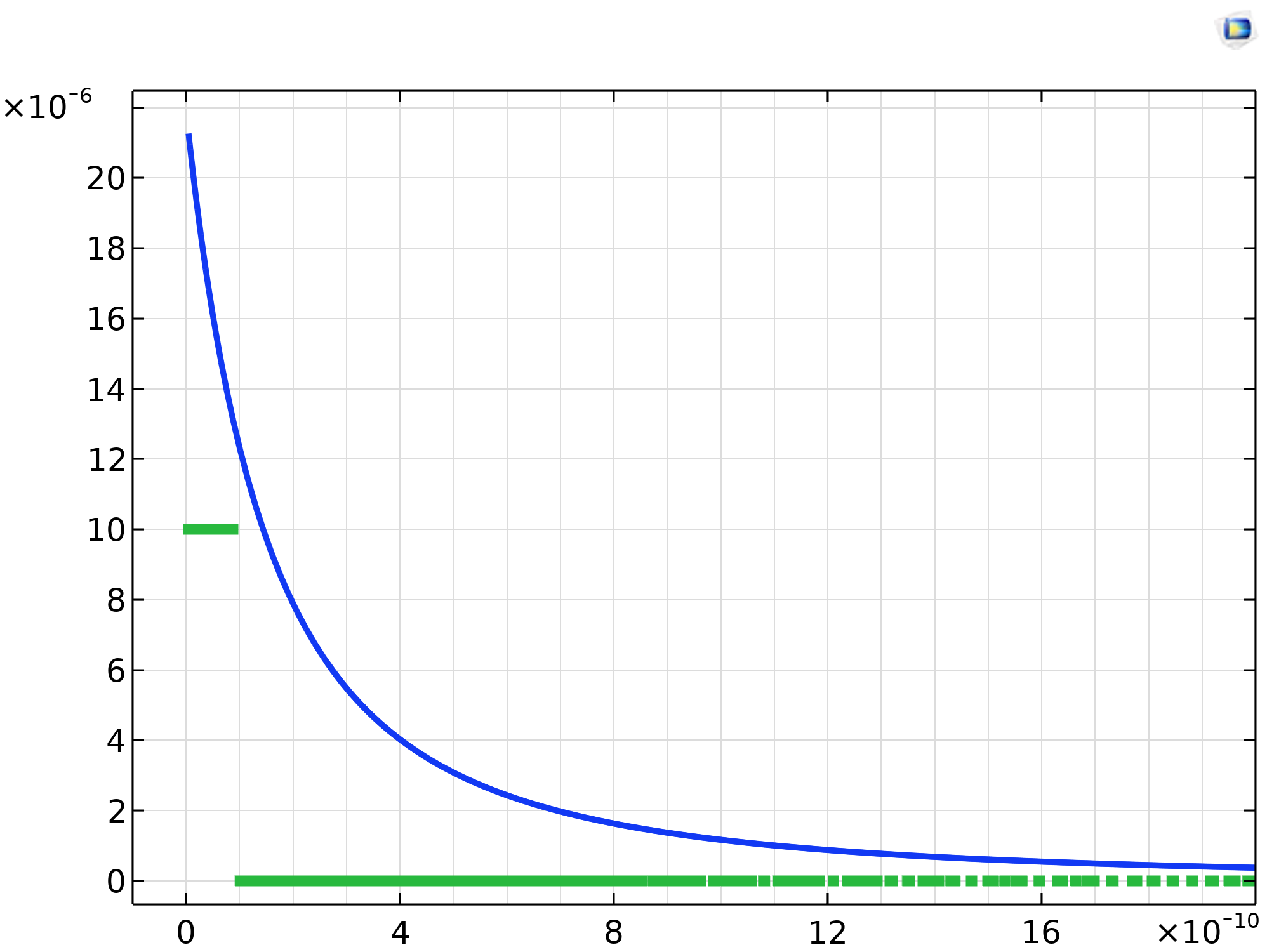
The expression used to calculate the sphere-hemisphere attraction described by the LJ potential is *Fv*dw = $\frac{\pi^{2}\rho^{2}AR}{6d^{2}}$, where *A* is the Hamaker constant for the studied interaction, that is in the order of 10^19^ J, *R* is the radius of the hemisphere and has a value of the pillars tip; *d* denotes the distance between the points of interaction, which varies during the simulation. ^[1]^ To analyze the interaction between the virus and the surface, we employed the stationary analysis of COMSOL’s contact method to define the equilibrium between the deformation of the two objects and their physical attraction. At every point of the virus’ surface, the force is determined by the distance to the pillars along the normal at that point. If the two surfaces are in contact, then the force becomes zero, representing the balance between the repulsive and attractive LJ potential. Additionally, adhesion makes sure no slip occurs below 0.25 nm; see **Figure S7** below.

**Figure S7.** Attraction force (blue) and adhesion activation (green) versus the distance between a virus and nanospikes.

In **Figure S7**, the blue curve represents the attraction force between the virus and the nanopillars that decreases quadratically with the increase of the distance. Also, we plot the adhesion denoted by green points. If at particular distance adhesion is activated, then a positive value corresponds to it, if not, the value is zero. The adhesion is defined to activate below 0.25 nm but as distance is also mesh dependent in the particular case it is activated at 0.1 nm. As the adhesion layer is defined by the elastic properties of the viral envelope, below 0.1 nm the envelope and the spikes are bonded together. This way we model a stick behavior when contact pairs are very close which switches to attraction somewhere between 0.1 nm and 0.25 nm. This way we are able to model deformation that is dependent on the distance between the boundaries of the envelope and the nanospikes.

To examine the sensitivity of the model to material properties, we varied the Young’s modulus of both the polymer substrate and the viral envelopes within ranges reported in the literature. Changing the substrate modulus from 1.2 to 3.6 GPa resulted in only slight decrease in the normalized strain (Figure S8a), consistent with the pillars being much stiffer than the virus. In contrast, increasing the viral capsid modulus from 50 to 200 MPa produced a clear decrease in localized strain (Figure S8b). Figure S8b shows that the stiffer capsids get easily destroyed. This is an outcome of their lower ability to cope with the shape of the pillars of the substrate. We have this explained in a way in the dedicated following subsection “Justification of inactivation threshold of viral envelope”. Importantly, these results confirm that the predicted mechano-virucidal response is robust across realistic material property ranges and is primarily governed by geometric confinement rather than fine-tuning of elastic constants.


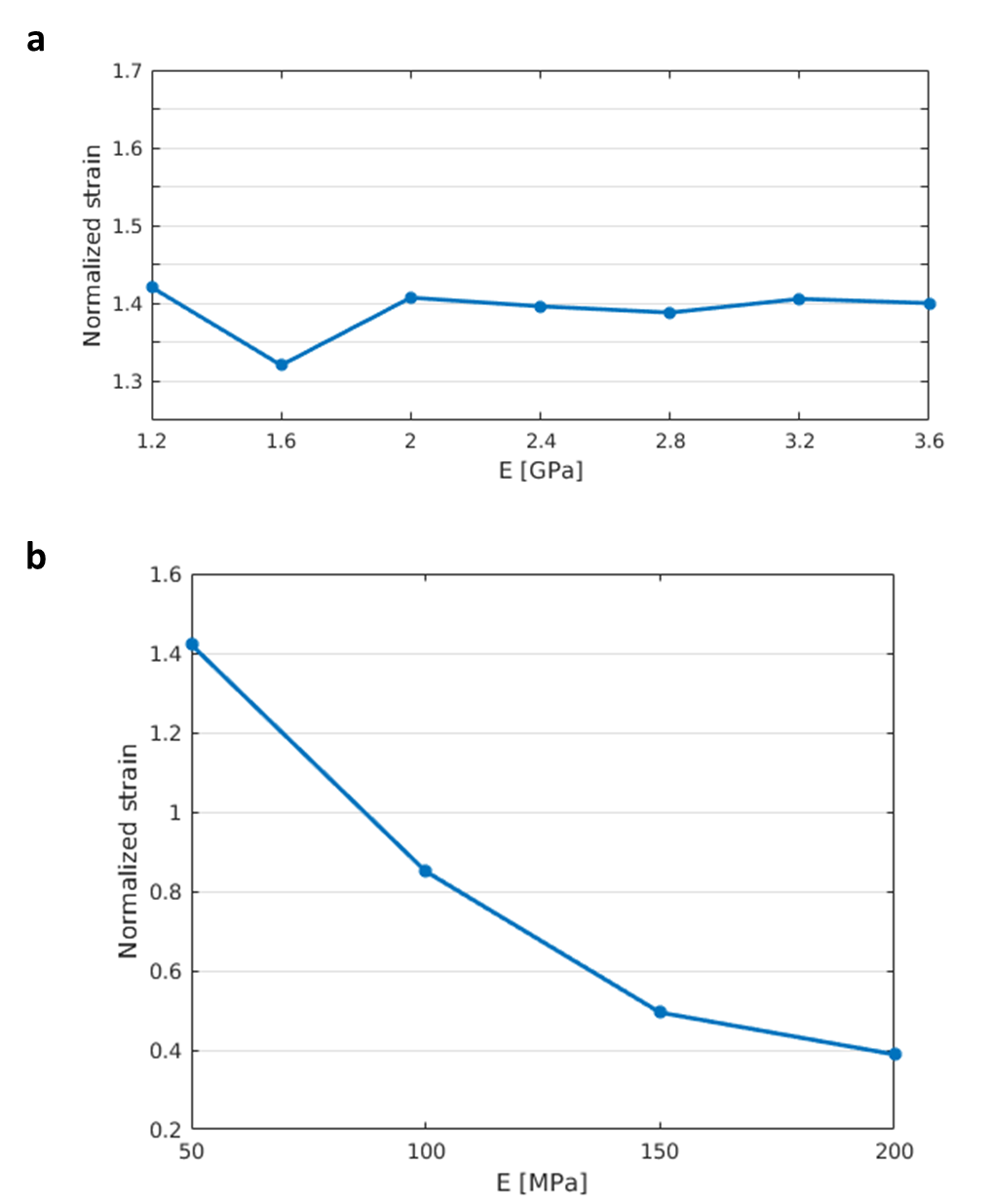


**Figure S8. Effect of material stiffness on the predicted mechano-virucidal response from FEM simulations.** (a) Normalized maximum strain in the viral envelope as a function of the Young’s modulus of the polymer substrate from 1.2 GPa to 3.6 GPa. Only a weak dependence is observed across this range, indicating that reasonable variations in substrate stiffness do not strongly influence the mechanical response. (b) Normalized maximum strain as a function of the Young’s modulus of the viral envelope (50–200 MPa). Considerable increase of the virucidal potential with the increase of the Young's modulus of the viral envelope from 50MPa to 200 MPa. Increasing capsid stiffness leads to a pronounced decrease in local strain, suggesting that stiffer viral particles are less able to conform to the nanopillar geometry and therefore experience higher mechanical stress. Normalization is performed using the same virucidal threshold as in Figure 5.

*Justification of inactivation threshold of viral envelope*

The values for the Young’s modulus and inactivation threshold of the viral envelope are critical to our FEM analysis, and we clarify our choices here. The stiffness of viral envelopes varies considerably, spanning an order of magnitude; for spherical enveloped viruses like hPIV-3, reported moduli range from approximately E = 6 MPa to above E = 1 GPa. ^[2-4]^ However, we observed that increasing the modulus from E = 50 MPa to E = 150 MPa in our simulations led to significant shifts, with maximum stress rising from 10 MPa to over 30 MPa for the same strain. Thus, by selecting a modulus of 50 MPa, we effectively model the lower boundary of possible envelope stiffness, representing more flexible viral envelopes that can undergo substantial strain before reaching the 10 MPa threshold associated with inactivation. The inactivation threshold of 10 MPa aligns with previous studies on viral envelopes, where damage was observed around this stress level. ^[5-8]^ Experimental data indicate that viral envelope disruption, as measured by AFM indentation tests, occurs at approximately 30% deformation relative to the virus diameter. This strain level corresponds to stresses between 36% [Klug] and 50% [Michel] of the Young’s modulus values they used in comparable simulations. ^[3, 9]^ These findings exceed the typical 10% strain-to-stress rule applied to bulk materials [Michel, Klug], thereby supporting our choice of a threshold of 20% ^[10]^ or 10 MPa for an E value of 50 MPa. This approach effectively models the resilience of flexible viral envelopes while ensuring that the selected parameters align with observed inactivation behavior.

*Antibacterial Assays*


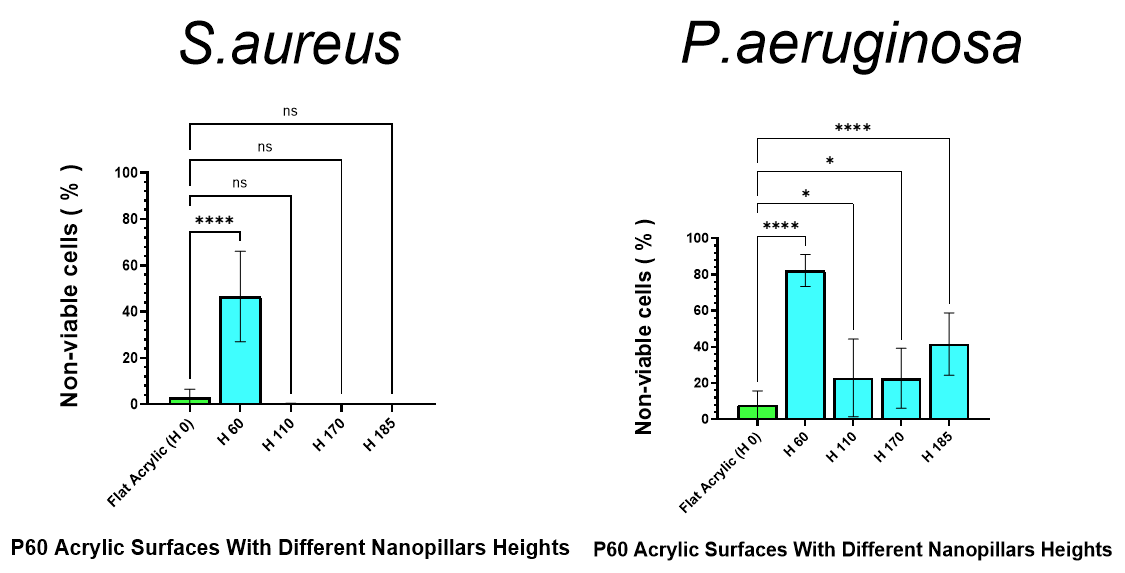


**Figure S9.** Evaluation of the bactericidal and antifouling performance of nanostructured acrylic surfaces (P 60) against *Staphylococcus aureus* (left) and *Pseudomonas aeruginosa* (right). Percentage of non-viable bacterial cells quantified from CLSM images and live/dead assay, against different heights of nanopillars (60, 110, 170 and 185 nm). Data are presented as mean ± standard deviation from independent experiments. Statistical significance is indicated as ***** *(p-value < 0.0001), ** (p-value < 0.01), * (p-value < 0.05),* and *ns (p-value > 0.05).*

*Additional SEM images*


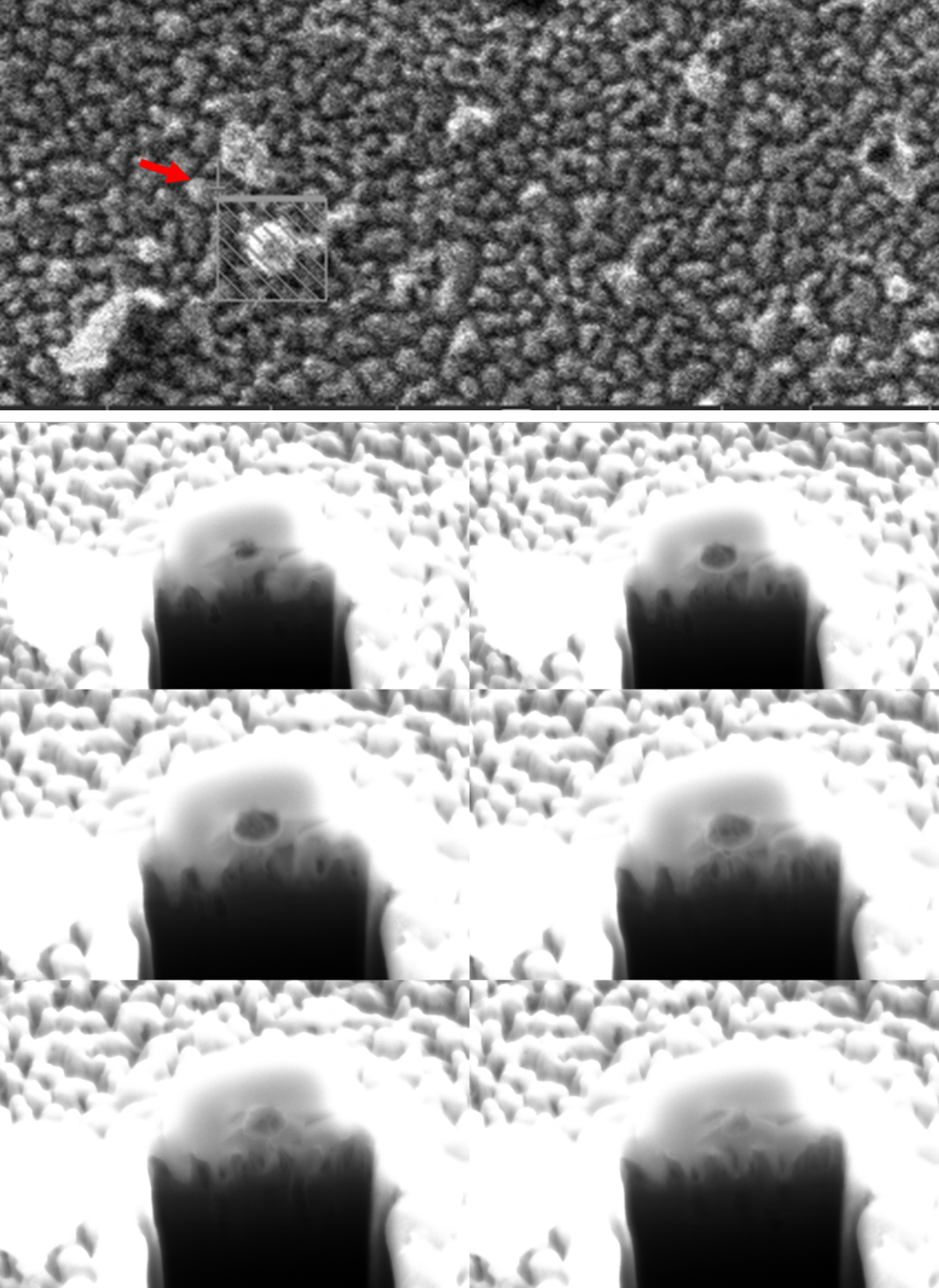


Figure **S10**. 60 nm stepwise FIB milling of hPIV-3 particles interacting with the P60_H185 nanostructured surface, showing progressive cross-sections through the virus–pillar interface. Scale bar is 500 nm.


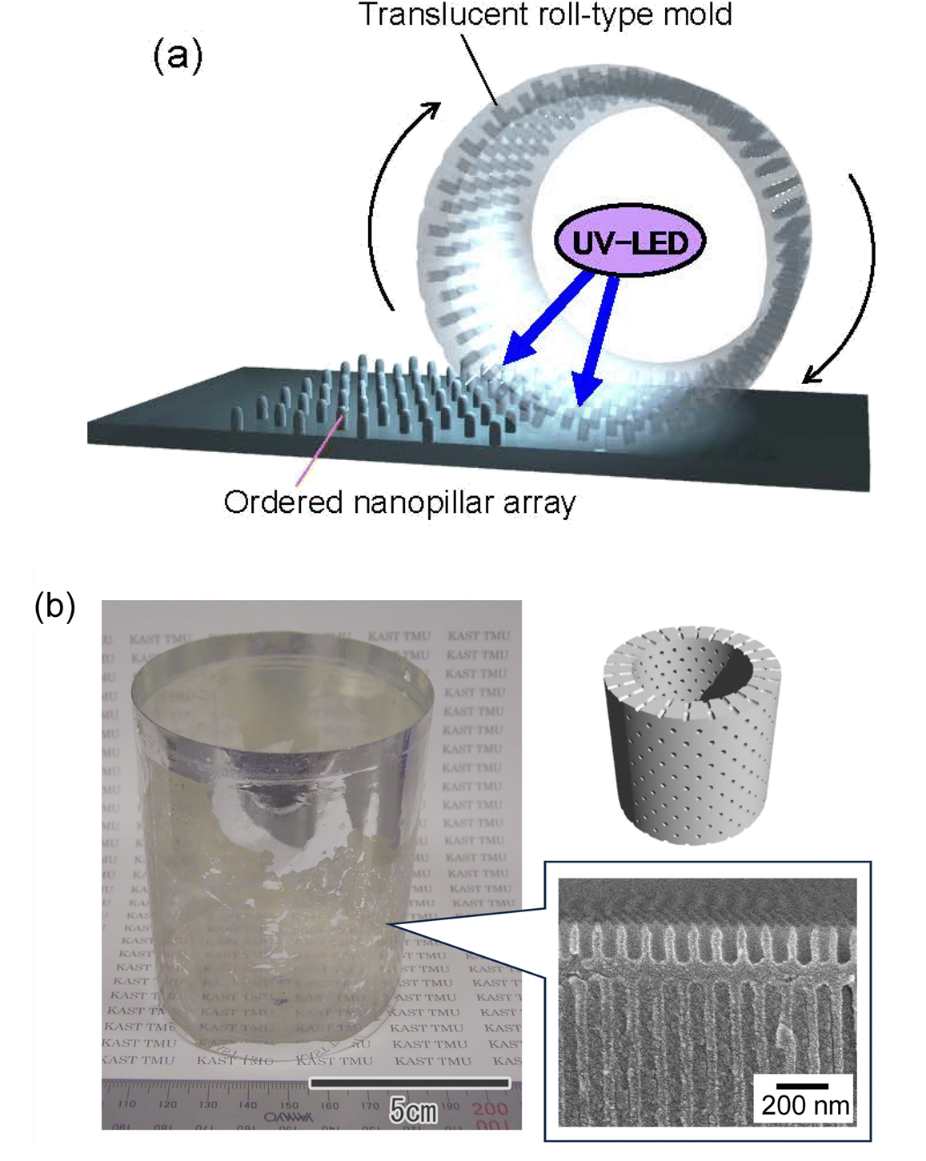


**Figure S11.** (a) Schematic illustration of continuous UV nanoimprinting using a roll-type translucent APA mold to generate ordered nanopillar arrays. (b) Photograph of the fabricated roll-type translucent APA mold (scale bar = 5 cm) and corresponding cross-sectional SEM image showing the nanopillar structures (scale bar = 200 nm). Figure adapted with permission from ^[11]^. Copyright © 2023 RSC Advances.

[
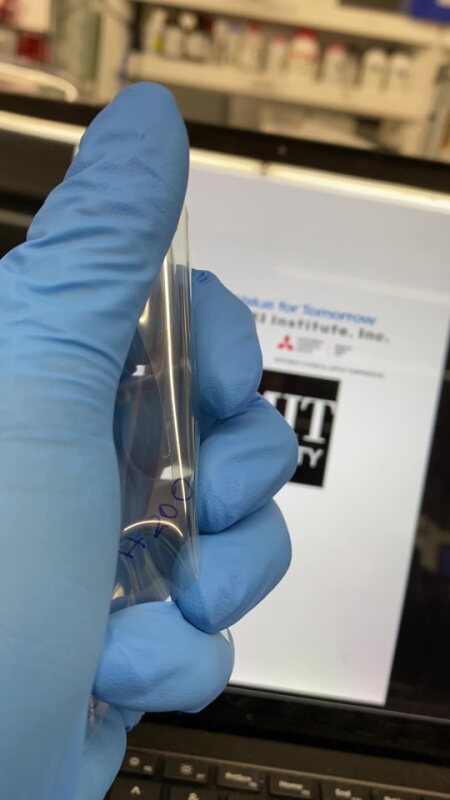
](https://rmiteduau-my.sharepoint.com/:v:/g/personal/s3909770_student_rmit_edu_au/ESX6APNXCF9Kvrbbn2tARsIBPBGNv1vx7_WdiWkvthbGzw?e=rgPoSf)

**Video S1**. Demonstration of the flexibility and optical transparency of the fabricated film.

**References**

[1] J. N. Israelachvili, *Intermolecular and Surface Forces (Third Edition)*, Academic Press, Boston **2011**.

[2] R. Zandi, D. Reguera, Mechanical Properties of Viral Capsids. Physical Review E **2005**, 72 (2), 021917

[3] J. P. Michel, I. L. Ivanovska, M. M. Gibbons, W. S. Klug, C. M. Knobler, G. J. L. Wuite, et al., Nanoindentation Studies of Full and Empty Viral Capsids and the Effects of Capsid Protein Mutations on Elasticity and Strength. Proceedings of the National Academy of Sciences **2006**, 103 (16), 6184

[4] D. P. Linklater, S. W. L. Mah, V. Tzanov, V. Baulin, N. A. Borg, G. Moad, et al., Current Perspectives on the Development of Virucidal Nano Surfaces. Current Opinion in Colloid & Interface Science **2023**, 67, 101720

[5] D. Gautam, N. Ahmed, V. K. P. Rao, Modelling the Effect of Geometry and Loading on Mechanical Response of Sars-Cov-2. BioNanoScience **2022**, 12 (3), 867

[6] S. Li, F. Eghiaian, C. Sieben, A. Herrmann, I. A. T. Schaap, Bending and Puncturing the Influenza Lipid Envelope. Biophys. J. **2011**, 100 (3), 637

[7] M. G. Mateu, Mechanical Properties of Viruses Analyzed by Atomic Force Microscopy: A Virological Perspective. Virus Res. **2012**, 168 (1), 1

[8] W. H. Roos, I. L. Ivanovska, A. Evilevitch, G. J. L. Wuite, Viral Capsids: Mechanical Characteristics, Genome Packaging and Delivery Mechanisms. Cell. Mol. Life Sci. **2007**, 64 (12), 1484

[9] M. M. Gibbons, W. S. Klug, Nonlinear Finite-Element Analysis of Nanoindentation of Viral Capsids. Physical Review E **2007**, 75 (3), 031901

[10] COMSOL Multiphysics®, Comsol Multiphysics Reference Manual Version 6.0. **2020**. <https://doc.comsol.com/6.0/docserver/#!/com.comsol.help.comsol/html_COMSOL_ReferenceManual.html> (accessed 2023-06-01).

[11] T. Yanagishita, N. Kumagai, H. Masuda, Flat and Roll-Type Translucent Anodic Porous Alumina Molds Anodized in Oxalic Acid for Uv Nanoimprint Lithography. RSC Advances **2023**, 13 (47), 33231
